# An algorithm for chemical genomic profiling that minimizes batch effects: bucket evaluations

**DOI:** 10.1186/1471-2105-13-245

**Published:** 2012-09-25

**Authors:** Daniel Shabtai, Guri Giaever, Corey Nislow

**Affiliations:** 1 Department of Cell and Systems Biology and the Donnelly Centre, University of Toronto, Toronto, ON M5S3E1, Canada; 2 Department of Pharmaceutical Sciences and the Donnelly Centre, University of Toronto, Toronto, ON M5S3E1, Canada; 3 Department of Molecular Genetics and the Donnelly Centre, University of Toronto, Toronto, ON M5S3E1, Canada

**Keywords:** Bucket evaluations, Batch effect, Chemogenomics, Correlation methods, Saccharomyces cerevisiae

## Abstract

**Background:**

Chemical genomics is an interdisciplinary field that combines small molecule perturbation with traditional genomics to understand gene function and to study the mode(s) of drug action. A benefit of chemical genomic screens is their breadth; each screen can capture the sensitivity of comprehensive collections of mutants or, in the case of mammalian cells, gene knock-downs, simultaneously. As with other large-scale experimental platforms, to compare and contrast such profiles, e.g. for clustering known compounds with uncharacterized compounds, a robust means to compare a large cohort of profiles is required. Existing methods for correlating different chemical profiles include diverse statistical discriminant analysis-based methods and specific gene filtering or normalization methods. Though powerful, none are ideal because they typically require one to define the disrupting effects, commonly known as batch effects, to detect true signal from experimental variation. These effects are not always known, and they can mask true biological differences. We present a method, Bucket Evaluations (BE) that surmounts many of these problems and is extensible to other datasets such as those obtained via gene expression profiling and which is platform independent.

**Results:**

We designed an algorithm to analyse chemogenomic profiles to identify potential targets of known drugs and new chemical compounds. We used levelled rank comparisons to identify drugs/compounds with similar profiles that minimizes batch effects and avoids the requirement of pre-defining the disrupting effects. This algorithm was also tested on gene expression microarray data and high throughput sequencing chemogenomic screens and found the method is applicable to a variety of dataset types.

**Conclusions:**

BE, along with various correlation methods on a collection of datasets proved to be highly accurate for locating similarity between experiments. BE is a non-parametric correlation approach, which is suitable for locating correlations in somewhat perturbed datasets such as chemical genomic profiles. We created software and a user interface for using BE, which is publically available.

## Background

Chemogenomics, the genome-wide analysis of the effects of chemical compounds, is a valuable approach to elucidate the mechanism of action of small molecules by identifying their cellular targets and target pathways [1]. Recent applications of chemical genomics in yeast include haploinsufficiency profiling and homozygote profiling of barcoded deletion collections [2-6], exploration of essential genes using temperature-sensitive mutants [7], molecular barcoded open reading frame libraries [8], decreased abundance by mRNA perturbation [9], multi-copy suppression profiling [10] and gene function and drug action analysis using the relationships between gene fitness profiles and drug inhibition profiles [11], to name a few.

We used chemogenomic profiles obtained from experiments that utilized the yeast *Saccharomyces cerevisiae* gene deletion collections [12], which include heterozygous and homozygous diploid deletions and haploid deletions. These screens measure growth of individual strains in a mixed population in the presence of diverse small molecules. In these screens, a decrease in the strain’s fitness can reflect that the deleted gene is the target of the chemical compound present (in heterozygous diploid deletion strains) or part of an affected pathway (homozygous diploid deletion strains).

In practice, a genome-wide chemical-genetic profile comprises the fitness of each strain relative to a mock treatment control profile. As each chemical compound produces a unique profile of gene sensitivities, comparing the profiles helps understand the similarity between the modes of action of compounds [13,14]. This “guilt-by-association” approach can suggest therapeutic applications for known compounds as well as the mode(s) of action of novel compounds [15,16]. Because most chemical profiles display a range of fitness defects, identifying similarities between chemical profiles requires a way to define similar fitness defect profiles. As part of this comparison, the method must emphasize those genes with highest fitness defect values, i.e. the strains most sensitive to treatment.

To analyze chemical genomics on a large scale (i.e. thousands-100 thousands of tests) a robust, extensible means to correct for variation is needed. This variation can come from many sources; including operator, laboratory, sample preparation and date [17,18]. Taken together, many profiles will cluster based on these non-biological parameters, into “batches”, which confounds any biological conclusions [19,20]. Furthermore, as throughput increases, and the method is adopted by different laboratories and platforms, batch effects will increase. These non-biological variation in results [18], are well recognized [21] and hinder the progress of 1) global analysis across different chemogenomic datasets and 2) efforts to integrate this data with orthologous genomic data. Although many batch effects [22] can be recorded for each experiment, one cannot account for all variation. One example of an effect that is not always recorded is the level of training, which varies over time, of the person performing the experiment. Another example is the temperature which affects all next generation sequencing experiments [23].

Due to batch effects, genomic profiles often display uninformative similarity according to these effects rather than the similarity of the underlying chemical biology [22,24]. Comparison algorithms, many of which do not consider batch effects, provide an inaccurate similarity mapping of profiles. Some algorithms require defining the variables that affects the results for an accurate comparison [22,24-27], yet these variables, and their relative impact are not always known.

To find similarity between experiments in a way that accommodates such uncertainty, we devised a method which finds correlation between experiments without the need to define the batch effects variables. This method is based on scaled ranks, which are scored according to a levelled scoring matrix, which provides a score for each gene-drug comparison. We evaluated the method using chemogenomic profiles (see methods), and compared the method to other existing correlation methods, including Pearson [28], Spearman [29], and Kendall [30] correlations, which also do not require prior knowledge of the variables that affect the results. Finally, we explored the extensibility of the Bucket Evaluations (BE) algorithm on other microarray data and barcode sequencing data (see results). By statistically evaluating results of the BE analysis compared to other correlation methods, we demonstrate its performance and illustrate its application to a variety of data types. We created software and a user interface, which is freely available such that the BE method can be applied for diverse experimental comparisons.

## Results and discussion

The BE algorithm is based on ranking and comparing a large number of columns within a dataset, and was initially applied to chemogenomic profiles. For a broader understanding of how the algorithm works, consider this analogy which equates chemogenomic profiles with spider habitats; There are over 40000 species of spiders living in a variety of habitats from hot deserts to artic regions [31]. Similar habitats should have similar groups of spider species, adapted to their environment. To evaluate similarity between spider habitats, one should compare the groups of successful (prosperous) species, rather than comparing the single most successful species because in very similar habitats A and B, the most successful species in A is not necessarily the most successful in B. A better way to measure habitat similarity is to ask, for example, if the most successful species in habitat A is, the top fifty most successful species in habitat B, because such a rank is still very high considering there are 40000 species.

**Figure 1:**
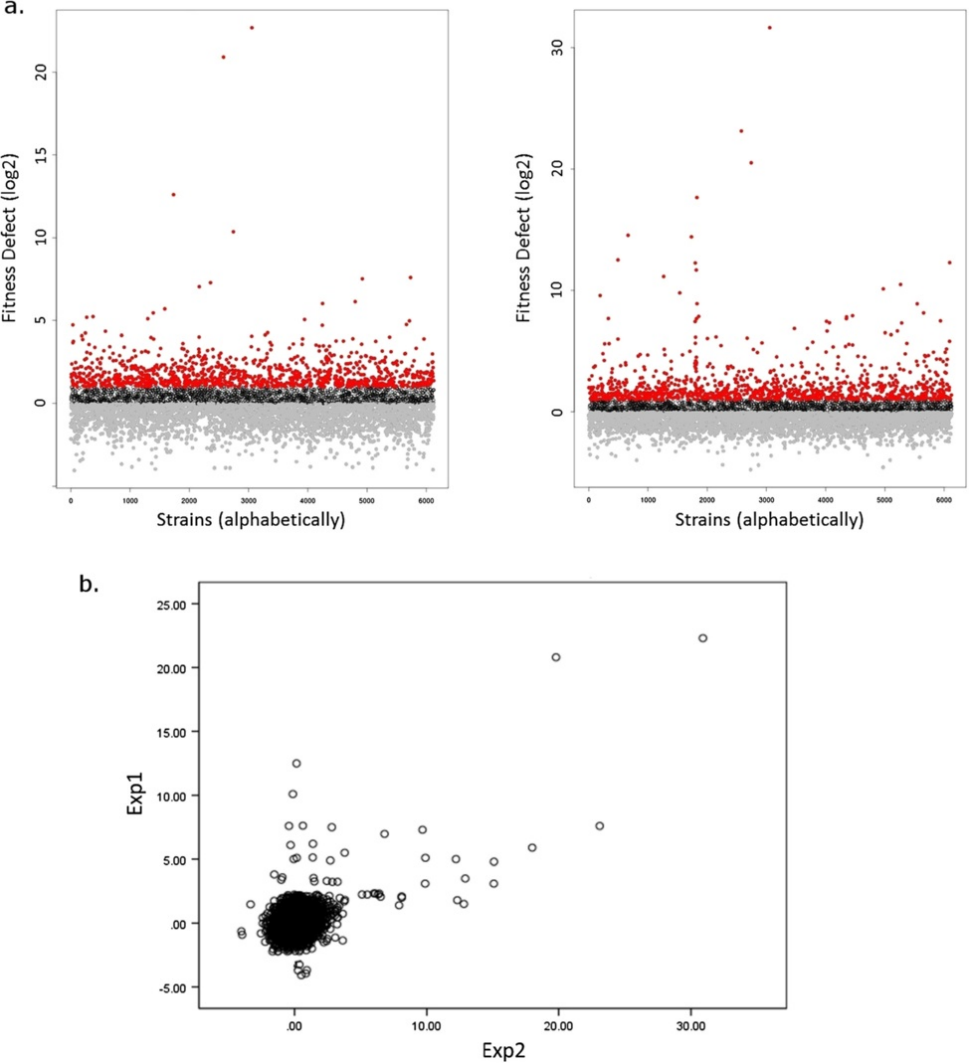
***Shabtai et al. Comparison of experiments performed on different dates.*** Two chemogenomic experiments performed using the same conditions (cantharidin, a protein phosphatase inhibitor) on different dates (**a**). These images show the extent of the differences between experiments that were performed using the same conditions. There is a difference in the scale of results (left experiment’s top value is ~22 representing a 10^6^ fold difference in abundance while right experiment’s top value is ~31 representing a 10^9^ fold difference in abundance). The lower results are the least affected genes, and include the majority of strains. These results vary in range of fitness defects between experiments, and are ignored because they are due to unmanageable differences between experiments, i.e. temperature perturbations. Despite the fact that the experiments were performed under the same conditions, the most sensitive deletion strains are not necessarily in the same ratio to each other nor are necessarily ranked in the same order (i.e. a strain can obtain the second highest fitness defect value in one experiment, and the third highest in another). Another representation of the differences between experiments is shown in image (**b**). The scatterplot shows an example of scores of two experiments performed using the same conditions. Top fitness defect scores are similar, though these strains are not in ranked the same for both experiments and have a different range of scores.

**Figure 2:**
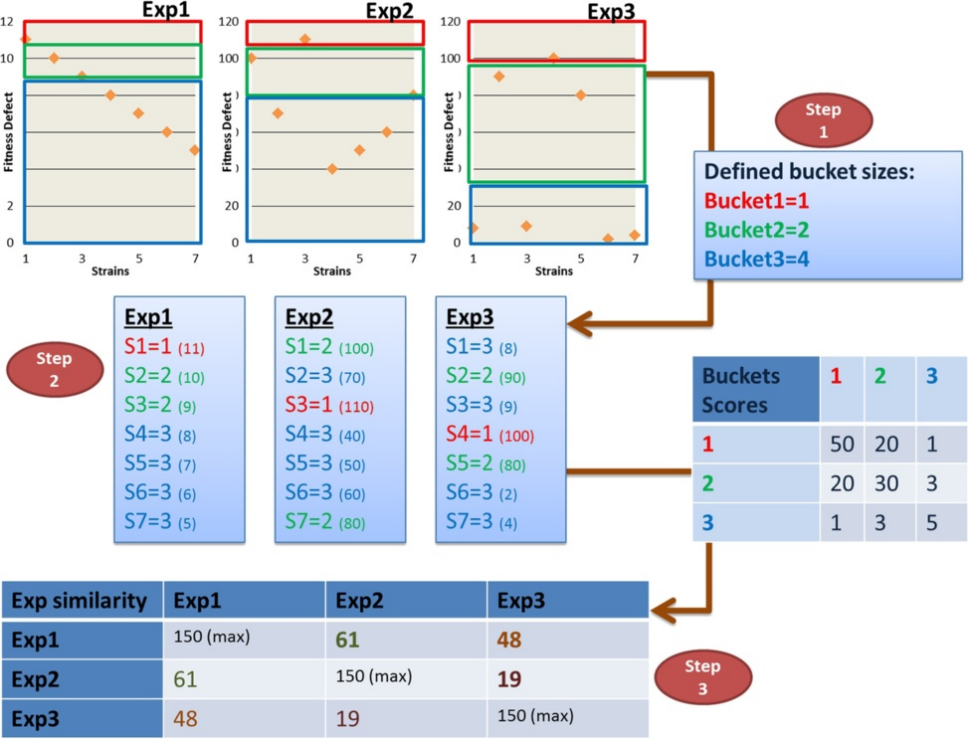
***Shabtai et al. Simplified example of BE implementation.*** Figure 2| A small-scale example of a basic implementation of BE for scoring experiments: (1) Define bucket sizes and scoring table values. (2) For each experiment, insert the strains in the relevant bucket according to rank. Each strain is mentioned with its bucket definition, while the values in brackets represent the fitness defect score. The fitness defect diagrams represent the buckets according to a coloured rectangle (red for bucket1, green for bucket2, and blue for bucket3). (3) Compare each experiment to the other experiments, and score similarity according to the scoring table. In this example, there is a higher similarity between Exp1-Exp3 rather than Exp2-Exp3. This example demonstrates that the BE algorithm gives greater emphasis to strains with a high fitness defect value rather than strains with a lower value.

Similar to the world of spiders, comparing the effect of chemical compounds requires examining the groups of genes affected by the chemical compounds rather than the top gene alone. There are many differences between profiles, such as scale of results, standard deviation, and a changing rank of gene values, even when the experiment was performed with the same compound at the same dosage, but on different days (Figure 1). These differences require analysing the ranking, not by comparing specific ranks, but by comparing groups of ranks. A pure rank comparison, meaning the highest value in one profile against the highest value in another profile and so on, gives poor results because it does not take into account the variability of ranks between genome-wide profiles. We addressed this problem in chemogenomic data using section comparisons, dividing each profile’s gene scores into sections, defined as buckets. The algorithm creates a weighted scoring system by ranking sections separately, and holding a higher score for highly ranked gene scores compared to lower ranked gene scores. Each section, or “bucket”, is defined as a subgroup of ranked scores, which are scored according to significance. The gene deletion strains with the highest fitness defect scores are considered the most significant for comparing profiles, as these deletion strains are the most influenced by the chemical compound. Therefore, we define the bucket sizes in each experiment according to significance, i.e. smaller buckets contain the most significant genes (genes with the higher fitness defects scores and lower fitness), whereas larger buckets contain the least significant genes (those with lower fitness defect scores and higher fitness). After the genes of each profile are parsed into buckets, we used a levelled scoring matrix (see methods) with weighted scores for scoring similarity between profiles, and evaluate a summed similarity score (Figure 2).

The levelled scoring matrix guidelines award a higher similarity score to genes located in lower buckets (e.g. when comparing two experiments, a gene located in bucket 2 for both experiments is awarded a higher score compared to a gene located in bucket 3 for both experiments), and to genes located in closer buckets (e.g. when comparing two experiments, a gene that is located in buckets 2 and 3 will get a higher score than a gene located in buckets 2 and 4). To implement the levelled scoring matrix guidelines, we devised a scoring matrix formula (Additional file 1: Table S1) which meets the requirements of the levelled scoring matrix (Additional file 2: Table S2, Additional file 3). These guidelines allowed us to find resemblance between profiles in addition to identifying profiles of repeated conditions.

### TAG4 barcode microarray dataset

**Figure 3:**
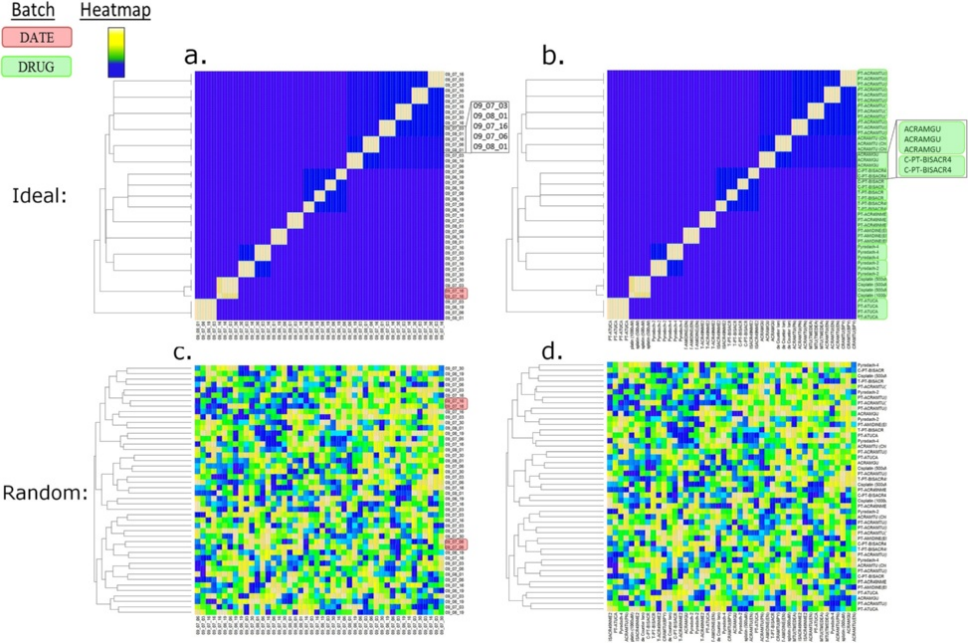
***Shabtai et al. Ideal and random outcome of TAG4 Microarray dataset cluster.*** Expected results of an ideal outcome and a random outcome. The left column displays the cluster of experiments where the labels are the dates on which the experiment was performed (**a, c**). Adjacent identical dates are displayed in a red rectangle to indicate when clustering occurs by date. The right column displays the cluster of experiments where the labels are the chemical compound that was used for each experiment (**b, d**). Adjacent identical chemical compounds are displayed in a green rectangle as shown in the legend, to indicate when the same chemical compounds are clustering together. The ideal result shows that experiments, performed using the same chemical compound, cluster together according to chemical compounds, where each cluster can be seen in a green rectangle (**b**). The ideal result also shows that the experiments cluster by date only when they were performed using the same chemical compound (**a**). The random score did not cluster any of the experiments according to chemical compound (**d**), and clustered experiments by date only by chance.

**Figure 4:**
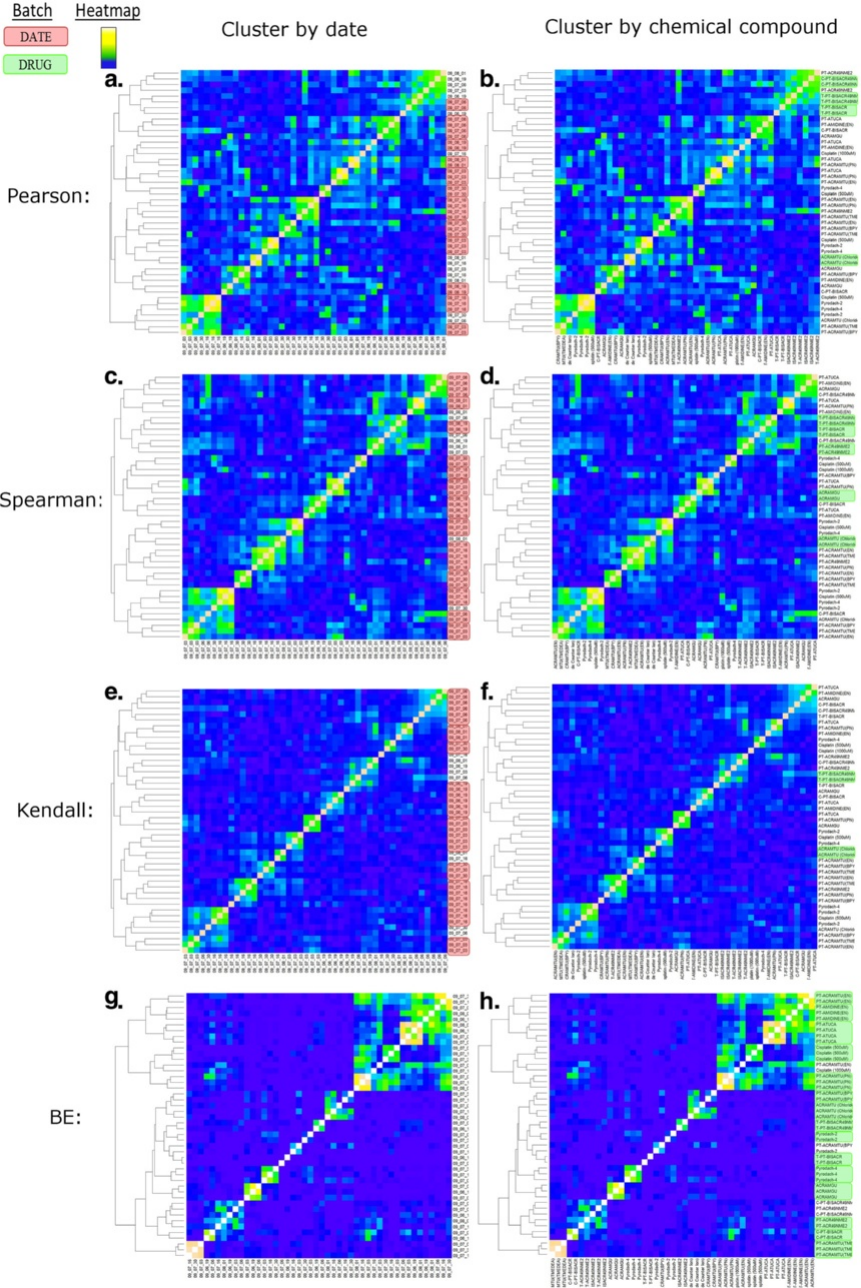
***Shabtai et al. Comparison of several correlation method outcomes using TAG4 Microarray dataset.*** Four correlation methods applied to the same dataset were clustered to show the performance of BE compared to other methods. The left column displays the cluster of experiments where the labels are the dates on which the experiment was performed (**a, c, e, g**). Adjacent identical dates are displayed in a red rectangle to indicate when clustering occurs by date. The right column displays the cluster of experiments where the labels are the chemical compound that was used for each experiment (**b, d, f, h**). Adjacent identical chemical compounds are displayed in a green rectangle to indicate when the same chemical compounds are clustering together. The desired result of a cluster is that similar conditions will cluster together. Examining the Pearson correlation cluster, the experiments cluster by date (**a**), due to a date batch effect. The BE method minimized the batch effect where identical dates did not cluster together (**g**), while identical conditions (chemical compounds) did cluster together (**h**).

**Figure 5:**
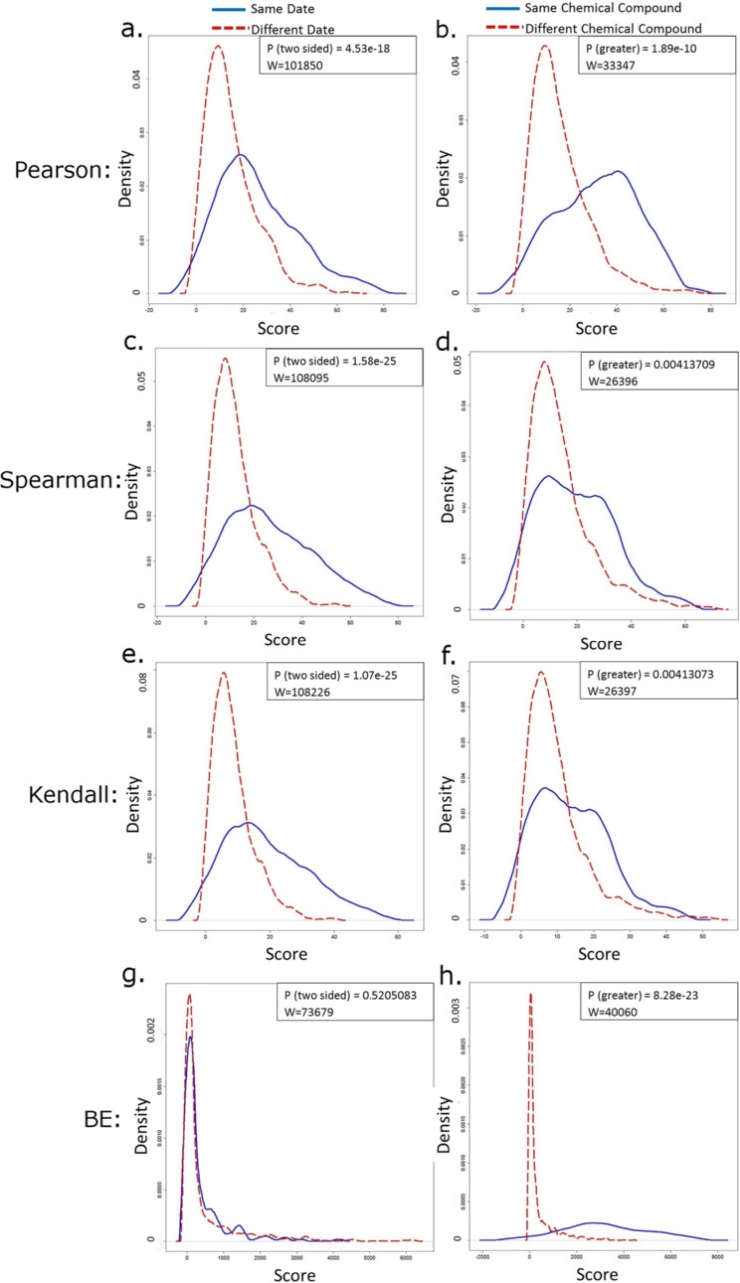
***Shabtai et al. Four correlation score distribution outcome of TAG4 Microarray dataset.*** The BE algorithm is least affected by the experiment date and most affected by experiment’s chemical compound used. The graphs show the distribution of scores. The graphs on the left column represent results affected by date (**a, c, e, g**). The solid blue line represents the score distribution of experiment pairs performed on identical dates, and the fragmented red line represents the score distribution of experiment pairs performed on different dates (**a, c, e, g**). The distributions according to date are significantly diverse for Pearson, Spearman and Kendall correlations (**a, c, e**), whereas the distributions by date are similar for BE correlation (**g**), meaning the scores were highly comparable for experiments done on the same date compared to experiments done on different dates. The graphs on the right column represent the score distributions affected by chemical compound (**b, d, f, h**). The solid blue line represents the score distribution of experiment pairs using identical chemical compounds, and the fragmented red line represents the score distribution of experiment pairs using different chemical compounds. All methods show that the distribution of the same chemical compound scores is significantly different than the distribution of different chemical compound scores, signifying, as expected, that all methods are affected by the chemical compound. The BE method shows the most significant difference in distribution compared to the other methods (**h**), being most affected by the chemical compound.

We ran the BE method on a dataset of TAG4 barcode microarray results (see methods), which included novel platinum based chemical compounds, in addition to well characterised compounds, such as cisplatin. The dataset was created by screening these compounds against a pool of ~6,000 barcoded deletion strains of *Saccharomyces cerevisiae*, 1200 essential genes as heterozygous diploids and 4800 non-essential genes as homozygous diploids to producing unique genome-wide profiles [2-5,32]. We used several correlation methods, including Pearson [28], Spearman [29] and Kendall [30], for finding similarities between the compound profiles. We then assessed their performance according to batching by date, an unwanted cluster outcome, versus batching by chemical compound, a desired cluster outcome (Figure 3, Figure 4). The results showed the BE method performed better than other methods, as measured by the statistical significance of the distribution of scores. We statistically assessed the distribution of similarity scores generated by each of the algorithms by using the Wilcoxon test (Figure 5) [33]. Typically, when clustering experiments to evaluate similarity, one would like to see experiments cluster according to experimental factors, i.e. chemical compound or mechanism of action, and not according to the date of the experiment, for example. To assess whether the date of the experiment had an effect in batching the scores, we used a two-sided Wilcoxon test on two vectors. The first vector contained the similarity scores of pairs of experiments performed on the same date, and the second vector contained scores of pairs of experiments performed on different dates. The graphs represent the distribution of similarity scores of both vectors (Figure 5a, 5c, 5e, 5g). These differences demonstrate a statistically significant shift in the distribution of scores between the two vectors when Pearson, Spearman or Kendall algorithms are used (p-values 10^-18^-10^-29^, Figure 5a, 5c, 5e), indicating a strong unwanted effect of the experiment’s date on the outcome. In contrast, the BE algorithm was not significantly affected by date (p > 0.05, Figure 5g). Indeed, the statistical evaluation confirmed that, compared to these other methods, the BE algorithm was least influenced by the date of the experiment, visualized as a highly similar distribution of scores for same dates and different dates. This is because BE compares groups of genes, rather than single gene ranks. (Figure 5g). We next evaluated whether the chemical compound used in an experiment had an effect in batching the scores, using the Wilcoxon test. We used two vectors: the first contained similarity scores for pairs of experiments performed with the same chemical compound, and the second contained scores of experiment pairs performed using different compounds (Figure 5b, 5d, 5f, 5h). Repeated experiments, using the same chemical compound, received higher similarity scores compared to experiments using different chemical compounds. The graphs represent the distribution of similarity scores of both vectors, and demonstrate a statistically significant shift in distribution for all algorithms used, indicating all methods used are affected by the chemical compound present. This was notable for the BE algorithm, which attained the lowest p-value (p = 8.28e-23, W = 40060) compared to the other methods (1.89e-10 < p < 0.0041, 26396 < W < 33347), confirming that the chemical compound has the strongest effect on the batching of scores rated by the BE method, and seen where the distribution of scores for different compounds is much lower than the distribution of scores for identical compounds (Figure 5h). To summarize this application of the BE algorithm, BE showed a clear difference in the distributions of scores between date and chemical compound, showing date has less effect on the BE method (Figure 5g), while chemical compounds have a strong effect on the BE method (Figure 5h). On the other hand, the differences in score distribution for each one of the correlation methods other than BE, look similar for both date and chemical compound, which means that experiments performed on the same date receive a score distribution nearly as high as experiments where the same chemical compound was used (Figure 5a-b, 5c-d, 5e-f).

### TAG3 microarray 2004 PNAS dataset

In order to evaluate the BE method on other types of datasets, we tested the method on a dataset which included 80 published microarray results for 10 different FDA approved drugs [3]. The assay used Haploinsufficiency Profiling, which comprises all 6200 diploid heterozygous yeast strains that can manifest sensitivity to compounds that inhibit the product of the heterozygous locus. This dataset consisted of 4 to 16 replicate experiments for each drug [4]. The BE algorithm successfully located similarity between drugs (Additional file 4: Table S3), recapitulating the previously reported similarity between three drugs: alverine-citrate, dyclonine, and fenpropimorph (Additional file 5: Figure S1d), demonstrating the accuracy of the algorithm [3]. In the original study, the similarity between drugs was found using a parametric method that set a threshold to ignore genes with low fitness defects (<3SD) [3], the BE method is non-parametric and did not ignore any genes for scoring similarity between experiments. We assessed the similarity results using other methods, including Pearson, Spearman and Kendall correlations, which all found similarity between these drugs. However, BE was the only method which found these three drugs as most similar to one another (Additional file 5: Figure S1). All methods found the replicate experiments as most similar to one another, scoring the drug itself within the top two most similar drugs.

### Gene expression (transcript abundance) dataset

Having shown BE works on barcode data from different studies, we next evaluated the BE method on an entirely different data type, genome-wide expression profiles from yeast. In this instance, gene expression is the measurement of transcript abundance, which is used as a proxy to measure the relative transcriptional activity of genes. Using microarrays, this process allows analyzing thousands of genes at once, providing a global picture of transcript abundance. For this analysis we selected the widely cited study of Gasch et al. which contains microarray results for 173 environmental stress experiments for all ~6000 genes [34]. This data was composed of gene expression abundances of *Saccharomyces cerevisiae* to diverse environmental conditions such as heat shock, oxidative and reductive stress, osmotic shock, nutrient starvation, DNA damage and extreme pH. In this dataset, high correlation scores between genes, represented by the transcript abundance measured, are indicative of a shared response to stress. These data were initially analyzed using fuzzy k-means [35], a method that differs from the standard k-means, as it provides a membership value for each gene to a centroid. Such membership permits each gene (scored according to transcript abundance) to belong to more than one centroid, which is critical because each gene may be co-regulated with several groups. Gasch and co-workers used prior knowledge about the data to select the k value according to the expected number of clusters, and chose the initial centroid locations according to known regulatory elements; we therefore used this analysis as a benchmark. The BE method positions the most affected genes, those with the highest score represented by transcript abundance, in the top significant buckets, providing a high score for comparing buckets among experiments with shared top genes, which resulted in a high correlation score specifically between groups of highly affected genes, confirming the previously reported group of ~900 specific genes which were found to be strongly affected throughout all stress treatments (Additional file 6: Figure S2). This group of environmental stress response genes represent a common gene expression response to stress [2]. The affected genes received statistically significant greater scores than the less affected genes where p < 2e-16 (Additional file 6: Figure S2c, Additional file 6: Figure S2f). These findings suggest that one can use the BE algorithm to locate unique groups of genes that display similar pattern of expression in certain experimental conditions, i.e. stress conditions or in the presence of chemical compounds. The BE method was found to perform as well as other correlation methods, which also display a significantly higher score for the reported genes (Additional file 7: Figure S3), including Pearson, Spearman and Kendall, for locating groups of similarly affected genes, presenting an additional application of the BE method.

### High throughput sequencing dataset

Next Generation Sequencing is rapidly being adopted and applied by applications previously dominated to microarrays, such as assessing abundance of yeast deletion strains using barcodes [36], full genome sequencing [37], transcriptome profiling [38,39] and epigenetics studies [40]. Accordingly, we evaluated the BE method on high throughput sequencing data of chemogenomic profiles performed in a manner similar to the barcode microarray data (see methods). For this method, the sequencing results consist of counts of barcode sequences representing the abundance of strains for each experiment [41]. The fitness defects are expressed as a log2 ratio of the strain specific barcode counts of strains grown in the present of a drug versus strains grown without the drug, for calculating the differences between the treatment and control. These results build a sequencing result matrix of strain fitness, a table of fitness defect scores for each strain in each experiment, that provided a dataset for using the BE. We ran the algorithm on 12 experiments which included 4 repeated experiments for each of 3 different drugs. The BE method successfully identified the experiments where repeated conditions clustered together according to the drug (Additional file 8: Figure S4a). Experiments performed using the same drug had a statistically significant higher scores than different drug experiments where P = 1.27e-20 (Additional file 8: Figure S4b). The BE method performed better than the Pearson correlation method (seen in cluster of repeated experiments in Additional file 9: Figure S5a compared to Additional file 9: Figure S5d), and as well as non-parametric methods including Spearman and Kendall correlations (Additional file 8: Figure S4, Additional file 9: Figure S5, Additional file 10: Figure S6). Such findings are significant as they confirm that one can use the BE method to compare different chemical compounds using data originated from high throughput sequencing experiments.

## Conclusions

Rigorous evaluations on several datasets, which included TAG4 microarrays, TAG3 microarrays, high throughput Barcode sequencing and gene expression microarrays, show that the BE algorithm overcomes most batch effects (Figure 4). We confirmed that the BE algorithm outperforms other well-established methods by statistically validating the differences of score distributions and comparing these differences between the BE method and other methods (Figure 5). Clustering of results showed the BE algorithm successfully identified similar conditions for microarray and sequencing data (Figure 4, Additional file 5: Figure S1d and Additional file 8: Figure S4). The BE method performed as well as other methods by successfully locating the group of key genes as most sensitive to environmental changes, attaining the highest similarity scores (Additional file 6: Figure S2).

Having tested the BE method on data collected from different technology platforms, we conclude that the method is applicable to other datasets where correlation between values is needed. For example, fine tuning the BE variables for different datasets, e.g. for high throughput sequencing data required modifying the first bucket size to be 0.05% of the total number of genes, and setting the maximum amount of buckets to 20 (Additional file 11: Figure S7). In general, achieving accurate correlation of results may involve similar fine-tuning. The general approach of bucket-weighted scores can therefore be applicable to both groups of highly similar profiles, and diverse matrices, according to the definition of the variables. This method may also be applicable to data collected from emerging technologies, such as new next generation sequencing applications, as finding correlation between results will continue to be beneficial [41].

We note that despite being applicable to many dataset models, like any algorithm it cannot satisfy all datasets. When considering whether to use the BE method or other methods, one should take into account several factors. First, whether the data is significant for both positive and negative values. As the BE method evaluates scores according to rank, datasets that are significant for both positive and negative values are not analyzed properly. This occurs due to negative values appraised as insignificant relative to positive values. For example, a genomic expression dataset can hold positive scores for induced genes and negative scores for repressed genes, represented by transcript abundance. Therefore both positive and negative values are significant, as they both show a change in cell response to the conditions measured in the experiment. One way to surmount this problem, which we used in our study, is to split the original dataset into two with the first dataset containing positive values, and the second containing only the absolute values of the original negative values. Running separate analysis for positive and negative values can then identify affected genes, represented by their transcript abundance.

The second factor is whether there is prior data that is relevant to the dataset which the user wishes to incorporate when assessing similarity between experiments. An example is the work done by Gasch and co-workers (see section 3.3), in which they wished to filter out highly regulated genes. Gasch and co-workers used the fuzzy k-means method, which uses prior knowledge about the expected number of clusters, and regulatory elements (see section 3.3). As a result many genes that are highly co-regulated, based on prior knowledge of the regulation factors, were filtered out. If the user wishes to ignore subsections of the dataset, the BE method is not suitable, as it is specifically designed to avoid the need of prior knowledge about the dataset, and to use an entire-dataset analysis approach.

We implemented the BE method so that it is available in a graphical user interface environment program. The application loads an input dataset, provided by the user, and produces a similarity matrix according to the BE variable definitions. The software is available for download (Additional files 12, and 13) along with sample input and output files (Additional files 14, 15, 16, and 17) [42].

## Methods

### Chemogenomic profiles

The chemogenomic profiles we compared were created by using the yeast *Saccharomyces cerevisiae* deletion strains collection [2-5]. Heterozygous and homozygous diploid gene deletion collections were used to determine those gene products of pathways most affected by treatment [12]. In this method each deletion strain is tagged with a barcode, which is a unique 20 bp sequence used for identification of the strain. After a collection of strains is grown in the presence of a compound, the sensitivity of each deletion strain is measured as a decrease in its abundance by PCR amplification of the strain specific barcodes followed by barcode microarray hybridization or barcode sequencing (Bar-Seq) [4,41]. This method allows identifying potential drug targets and/or genes and pathways required for growth in the presence of a compound [3,12].

The results of each experiment are microarray signal intensities or barcode sequence counts, which reflect barcode abundance and, by extrapolation, strain abundance. These values are normalized by evaluating the log2 ratio between the signal intensities of drug-treated pools and control pools, which are mock treated with DMSO. This value is represented as the strain’s fitness defect. In a typical experiment, a few strains show a high fitness defect while the majority show little or no defect relative to the control treatment. Lower values may be true sensitive strains, yet are not necessarily located when using a set threshold, because they are concealed within midrange values that are considered background.

### Levelled scoring matrix

The levelled scoring matrix is constructed of decreasing scores, from high scores for a gene in closely ranked groups (buckets) to low scores for a gene in distant groups (buckets). When comparing profiles, the score matrix yields the score of *S*_*i,j*_ to a gene located in bucket *i* and bucket *j* n each of the profiles compared. For a score of *S*_*i,j*_ the scoring matrix follows these guidelines: (1) For each experiment, the strains are divided into buckets. The buckets are ordered in ascending importance so that a lower bucket holds the strains with the highest fitness defect. (2) Assign higher scores for “hits” in different experiments which fall within the same bucket, while taking into consideration that first buckets are more significant than last buckets, where *S*_*i,j*_ for experiments *Exp*1 and *Exp*2, is the score of a fitness defect strain which is located in bucket *i* in *Exp*2, and in bucket *j* in *Exp*2. (3) $\forall i,j|i<j\Rightarrow S_{i,i}>S_{j,j}$ For example: *S*_1,1_ > S_1,2_. (4) Assign a higher score for hits in closer buckets: $\forall i,j,k|i<j<k\Rightarrow S_{i,j}>S_{i,k}$. For example: *S*_2,3_ > S_2,4_.

We built the scoring matrix formula, in accordance to these guidelines (Additional file 1: Table S1), where *n* represents the total number of buckets; *c* represents the current bucket column. The top score (bucket 1 vs. bucket 1) is a value set according to the total number of buckets, in order to achieve a wide spread of scores throughout the table. For example, the range of scores for n = 5 buckets is from *S*_1,5_ = 2.1 10^-4^ to *S*_1,1_ = 2^(5-1)^ = 16, while the range of scores for 11 buckets is from *S*_1,11_ = 9.9 10^-16^ to *S*_1,1_ = 2^(11-1)^ = 1024 (Additional file 2: Table S2). This example shows how the most significant buckets hold few genes (buckets are smaller in size), yet have the potential of receiving the highest scores giving more significance to the most sensitive genes, providing that the most sensitive genes appear in close buckets for both experiments being compared (such as the scores in the fragmented red rectangle). If a gene is in distant buckets, the score is lower, i.e. a strain in bucket 6 in both experiments is scored 1.42, while a strain in bucket 6 in one experiment, and in bucket 5 in another is scored 0.237 (Additional file 2: Table S2). For hits in the same bucket, the score will be more significant for a lower bucket, i.e. a strain in bucket 2 in both experiments will get a score of 512, while a strain in bucket 4 in both experiments will get a score of 42.67 (Additional file 2: Table S2).

By creating a general formula, rather than an unchangeable scoring matrix that corresponds to the guidelines, we allow flexibility in algorithm analysis for different types of data. Other scoring matrices, which correspond to the defined guidelines, may also be suitable. The formula we constructed allows defining the bucket sizes – how many genes can a bucket contain. If the most significant buckets contain fewer genes, the similarity score will be more stringent, and will provide a high similarity scores for experiments sharing few hits. When the most significant buckets contain many genes, the similarity score will be broader, and will find high similarity between experiments with larger distances between gene rank locations. The accompanying software provides the user with the ability to change these parameters according to the dataset, and in addition, using pre-set values for evaluating the suitable parameter values.

### Software imaging and implementation

Images and analysis were created using R [43]. Figure 1b was created using SPSS [31]. The BE software was developed using C# .NET 3.0 Framework. The software is available for download [42].

## Abbreviations

Bar-Seq: Barcode sequencing; BE: Bucket evaluations; DMSO: Dimethyl sulfoxide; DNA: Deoxyribonucleic acid; FDA: Food and drug administration; mRNA: Messenger ribonucleic acid; SD: Standard deviation.

## Competing interests

The authors declare that they have no competing interests.

## Authors’ contributions

DS designed and implemented the BE algorithm. CN GG and DS selected the datasets for testing the algorithm. CN and GG oversaw the project and edited the manuscript. The supplementary software architecture and implementation is credited to DS. All authors read the final manuscript.

## Supplementary Material

Additional file 1: Table S1 *Shabtai et al.* Scoring matrix formula. A scoring matrix formula in accordance to the guidelines needed for BE scoring. The top score (bucket 1 vs. bucket 1) depends on the total number of buckets (n) in order to achieve a wide spread of scores throughout the table. For example, the range of scores for n = 5 buckets is from *S*_1,5_ = 2.1 10^-4^ to *S*_1,1_ = 2^(5-1)^ = 16, while the range of scores for 11 buckets is from *S*_1,11_ = 9.9 10^-16^ to *S*_1,1_ = 2^(11-1)^ = 1024 (as seen in Additional file 2: **Table S2**). n = Total number of buckets. c = Current bucket column *S*_i,j_= Score for when comparing bucket *i* to bucket *j*. Click here for file

Additional file 2: Table S2 *Shabtai et al.* Implementation example of the scoring matrix. Implementation example of the scoring matrix (Additional file 1: **Table S1**) where the number of buckets (n) equals 11 (therefore *S*1,1 = 2^(*n*-1)^ = 1024). The cell colour, ranging from yellow to blue, indicates the significance of a similarity score when comparing gene ranks between experiments. The most significant buckets hold few genes (buckets are smaller in size), yet have the potential of receiving the highest scores (shown in blue) giving more significance to the most sensitive genes, providing that the most sensitive genes appear in close buckets for both experiments being compared (such as the scores in the fragmented red rectangle). If a gene is in different buckets for the compared experiments, the score is lower, i.e. a strain in bucket 6 in both experiments is scored 1.42, while a strain in bucket 6 in one experiment, and in bucket 5 in another is scored 0.237. For hits in the same bucket, the score will be more significant for a lower bucket, i.e. a strain in bucket 2 in both experiments will get a score of 512, while a strain in bucket 4 in both experiments will get a score of 42.67. Click here for file

Additional file 3 **Scoring Matrix Example.** An Excel file which implements the scoring matrix formula (Additional file 1: **Table S1**), and shows how each score is calculated (see formula bar of each cell). Click here for file

Additional file 4: Table S3 *Shabtai et al.* Top three similar drugs in TAG3 Microarray dataset using several correlation methods. Top three drug similarity scores of the group of drugs that were reported as similar. Each drug column mentions the amount of drugs that were in the top three highest scores. For example, Pearson correlation showed alverine-citrate experiments as most similar to all three reported drugs: alverine-citrate, dyclonine and fenpropimorph. BE is the only method which identified the similarity for all drugs (100%) recapitulating the previously reported similarity of alverine-citrate, dyclonine and fenpropimorph. Click here for file

Additional file 5: Figure S1 *Shabtai et al.* Comparison of TAG3 Microarray similarity results. A comparison of barcode TAG3 microarray similarity results between a variety of correlation methods including Pearson **(a)**, Spearman **(b)**, Kendall **(c)** and BE **(d)**. Each colour represents a drug, and each column represents similarity scores of one drug to other drugs using coloured bars according to the compared drug. An example of a column is seen in figure a showing similarity levels to alverine citrate as calculated using Pearson correlation. Each bar represents a different drug, and the size of each bar represents the level of similarity to alverine citrate as a percentage of the top score of the method used **(e)**. To recapitulate the previously reported similarity between three drugs: alverine-citrate, dyclonine, and fenpropimorph, we used different methods, and ascertained all methods found similarity between these drugs as seen in the orange (alverine-citrate), green (dyclonine) and blue (fenpropimorph) bars. The top three most similar drugs are mentioned within the drug’s similarity column of each method, in a rhombus, for these drugs. For the BE method, the top three values for these compounds are the three compounds themselves, where the chemical structure of these drugs is similar explained by a similar mode of action (d). BE was the only method where all three drugs shared the same top three similar drugs. Click here for file

Additional file 6: Figure S2 *Shabtai et al.* Gene similarity results using BE on a Genomic Expression Dataset. In order to locate genes of interest, the BE method was executed on a dataset of yeast response to environmental changes. Because both negative values and positive values are meaningful, we created two datasets where one included all positive values (negative values were set to 0) and the second dataset included all negative values, set to their absolute value (positive values were set to 0). Results show how the BE method successfully located the most affected genes , according to measured transcript abundance, confirming the 586 positively affected genes **(a)**, and the 282 negatively affected genes **(d)**, marked in yellow in the ranked scores as seen as the exceedingly affected genes. The higher scores, that the 868 genes received compared to other genes, can be seen in light green for both positive **(b)** and negative **(e)** scores. The 868 genes received statistically significant greater scores than other genes both for positive (c P<2e-16) and negative (f P<2e-16) affected genes where the full green line represents the positively (c), induced genes **(c)**, and negatively, repressed genes **(f)**, and the fragmented red line represents the rest of the genes. The distribution of scores for the less affected genes displays two peaks due to lower scores for the negative genes compared to the other genes and seen as two dark stripes **(b)**, marked in blue at the low end scores **(a)**. Click here for file

Additional file 7: Figure S3 * Shabtai et al.* Score distributions of several comparison methods for a Genomic Expression dataset. The distribution of scores of the Gasch et al. study dataset. The green line represents the score distribution of the previously reported group of genes found to be significantly affected by the stress treatments. For the negative score dataset **(a, b, c, d)**, the green line represents the group of ~300 repressed genes, and for the positive score dataset **(e, f, g, h)**, the green line represents the group of ~600 induced genes. The fragmented red line represents the score distribution of the genes other than the reported group of genes. The methods used for comparing the score distribution included BE, Pearson, Spearman and Kendall correlations. All methods showed there are statistically significant higher scores for the reported genes (similar W statistic value) successfully locating the affected genes. The BE method performed as well as other methods identifying the affected group of genes, moreover, it differentiated the lower results and identified anti-correlation between the two groups of ~300 and ~600 affected genes by showing two peaks for the lower scores. Click here for file

Additional file 8: Figure S4 *Shabtai et al.* Similarity results between experiments using BE on a sequencing dataset. Running the BE method on high throughput sequencing data successfully cluster experiments using the same drug **(a)**. We used the Wilcoxon test to evaluate the distribution of the scores **(b)** of same drug experiment scores (green line) and different drug experiment scores (red line). These results showed that same drug scores received a statistically significant higher score than different drug scores (P=1.27e-20). Click here for file

Additional file 9: Figure S5 *Shabtai et al.* Comparison of several correlation method outcomes using TAG4 Microarray dataset. A comparison of several methods, including Pearson **(a)**, Spearman **(b)**, Kendall **(c)** and BE **(d)**, for finding correlations between barcode sequencing experiments. A heat-map and dendrogram displays the clustering of experiments for each method. For BE, Spearman and Kendall methods, all experiments that were performed using the same drug clustered together, showing BE **(d)** performed as well as other non-parametric methods, including Spearman **(b)** and Kendall **(c)**. BE performed better than the Pearson correlation **(a)**, where not all same-drug experiments clustered together. Click here for file

Additional file 10: Figure S6 *Shabtai et al.* Score distributions of several comparison methods for a sequencing dataset. The score distribution of several methods, including Pearson **(a)**, Spearman **(b)**, Kendall **(c)** and BE **(d)** of correlations scores of barcode sequencing experiments. The full green line represents the similarity score distribution of experiments performed using the same drug, while the fragmented red line represents the score distribution of experiments performed using different drugs. All methods present statistically significant greater scores to experiments performed using the same drug. Click here for file

Additional file 11: Figure S7 *Shabtai et al.* Fine tuning the BE variable values. The output of using different BE variable values for high throughput sequencing dataset shows how fine tuning the value can produce a better result. When using an initial bucket size of 5%, not all experiments cluster according to the chemical compound **(a)**. When using an initial bucket size of 0.05%, all experiments cluster according to the chemical compound **(b)**, showing how fine tuning the value can produce better results. Click here for file

Additional file 12 **Bucket Evaluations Software.** An executable file of the BE software. Click here for file

Additional file 13 **Software Manual.** An explanation of the software architecture, and how to use the software. Click here for file

Additional file 14 **Sample Input Dataset.** An example dataset of an input file for using the software (12 experiments, 6003 genes). Click here for file

Additional file 15 **Sample Output Stringent.** The file produced when running the sample input, comparing the columns (experiments) using stringent pre-set values. Click here for file

Additional file 16 **Sample Output Intermediate.** The file produced when running the sample input, comparing the columns (experiments) using intermediate pre-set values. Click here for file

Additional file 17 **Sample Output Broad.** The file produced when running the sample input, comparing the columns (experiments) using broad pre-set values. Click here for file
