# Supplementary figures and images for: An algorithm for chemical genomic profiling that minimizes batch effects: bucket evaluations
Source: BMC Bioinformatics. 2012 Sep 25;13:245. doi: 10.1186/1471-2105-13-245 (PMC3780717; doi:10.1186/1471-2105-13-245)

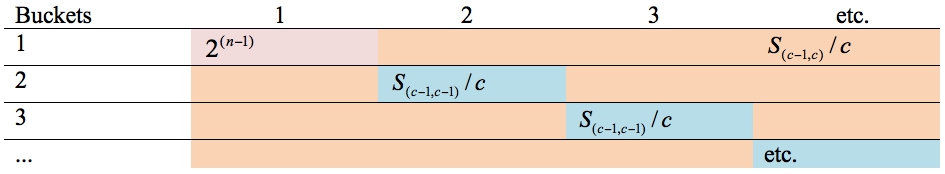

Supplement: Additional file 1: Table S1 — Shabtai et al. Scoring matrix formula. A scoring matrix formula in accordance to the guidelines needed for BE scoring. The top score (bucket 1 vs. bucket 1) depends on the total number of buckets (n) in order to achieve a wide spread of scores throughout the table. For example, the range of scores for n = 5 buckets is from S1,5 = 2.1 10-4 to S1,1 = 2(5-1) = 16, while the range of scores for 11 buckets is from S1,11 = 9.9 10-16 to S1,1 = 2(11-1) = 1024 (as seen in Additional file 2: Table S2). n = Total number of buckets. c = Current bucket column Si,j= Score for when comparing bucket i to bucket j. [file 1471-2105-13-245-S1.jpg]

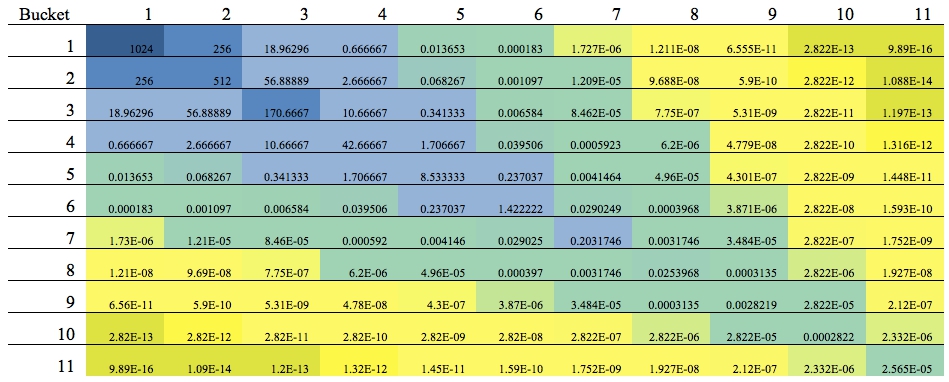

Supplement: Additional file 2: Table S2 — Shabtai et al. Implementation example of the scoring matrix. Implementation example of the scoring matrix (Additional file 1: Table S1) where the number of buckets (n) equals 11 (therefore S1,1 = 2(n-1) = 1024). The cell colour, ranging from yellow to blue, indicates the significance of a similarity score when comparing gene ranks between experiments. The most significant buckets hold few genes (buckets are smaller in size), yet have the potential of receiving the highest scores (shown in blue) giving more significance to the most sensitive genes, providing that the most sensitive genes appear in close buckets for both experiments being compared (such as the scores in the fragmented red rectangle). If a gene is in different buckets for the compared experiments, the score is lower, i.e. a strain in bucket 6 in both experiments is scored 1.42, while a strain in bucket 6 in one experiment, and in bucket 5 in another is scored 0.237. For hits in the same bucket, the score will be more significant for a lower bucket, i.e. a strain in bucket 2 in both experiments will get a score of 512, while a strain in bucket 4 in both experiments will get a score of 42.67. [file 1471-2105-13-245-S2.jpg]

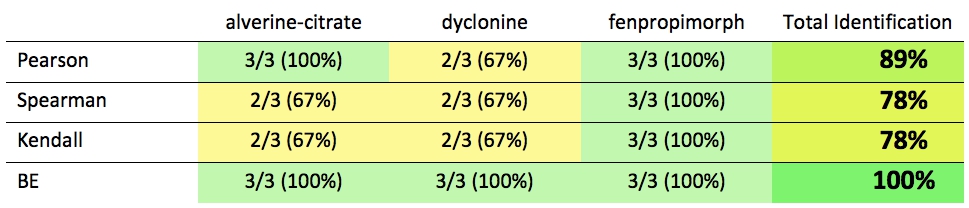

Supplement: Additional file 4: Table S3 — Shabtai et al. Top three similar drugs in TAG3 Microarray dataset using several correlation methods. Top three drug similarity scores of the group of drugs that were reported as similar. Each drug column mentions the amount of drugs that were in the top three highest scores. For example, Pearson correlation showed alverine-citrate experiments as most similar to all three reported drugs: alverine-citrate, dyclonine and fenpropimorph. BE is the only method which identified the similarity for all drugs (100%) recapitulating the previously reported similarity of alverine-citrate, dyclonine and fenpropimorph. [file 1471-2105-13-245-S4.jpg]

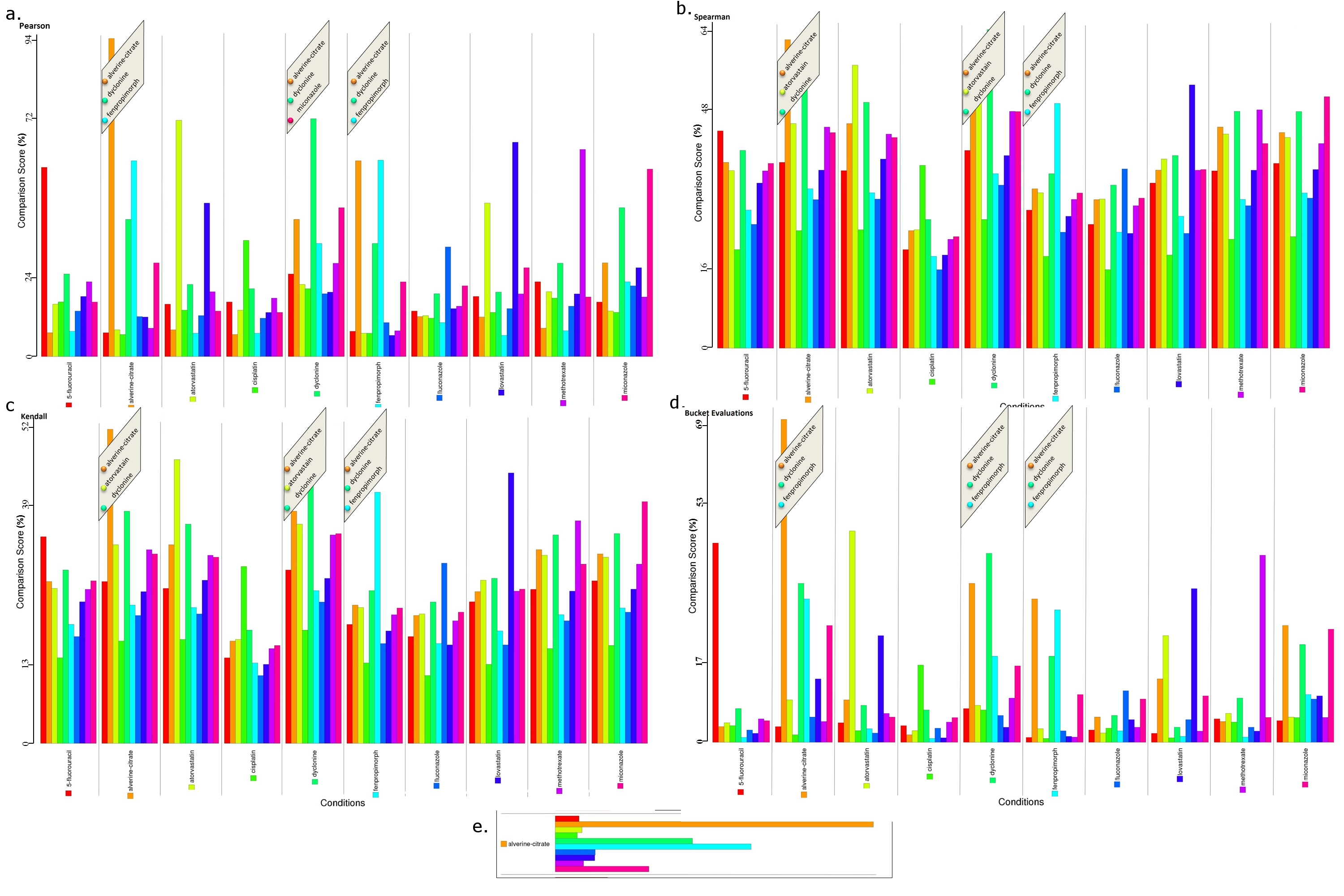

Supplement: Additional file 5: Figure S1 — Shabtai et al. Comparison of TAG3 Microarray similarity results. A comparison of barcode TAG3 microarray similarity results between a variety of correlation methods including Pearson (a), Spearman (b), Kendall (c) and BE (d). Each colour represents a drug, and each column represents similarity scores of one drug to other drugs using coloured bars according to the compared drug. An example of a column is seen in figure a showing similarity levels to alverine citrate as calculated using Pearson correlation. Each bar represents a different drug, and the size of each bar represents the level of similarity to alverine citrate as a percentage of the top score of the method used (e). To recapitulate the previously reported similarity between three drugs: alverine-citrate, dyclonine, and fenpropimorph, we used different methods, and ascertained all methods found similarity between these drugs as seen in the orange (alverine-citrate), green (dyclonine) and blue (fenpropimorph) bars. The top three most similar drugs are mentioned within the drug’s similarity column of each method, in a rhombus, for these drugs. For the BE method, the top three values for these compounds are the three compounds themselves, where the chemical structure of these drugs is similar explained by a similar mode of action (d). BE was the only method where all three drugs shared the same top three similar drugs. [file 1471-2105-13-245-S5.jpg]

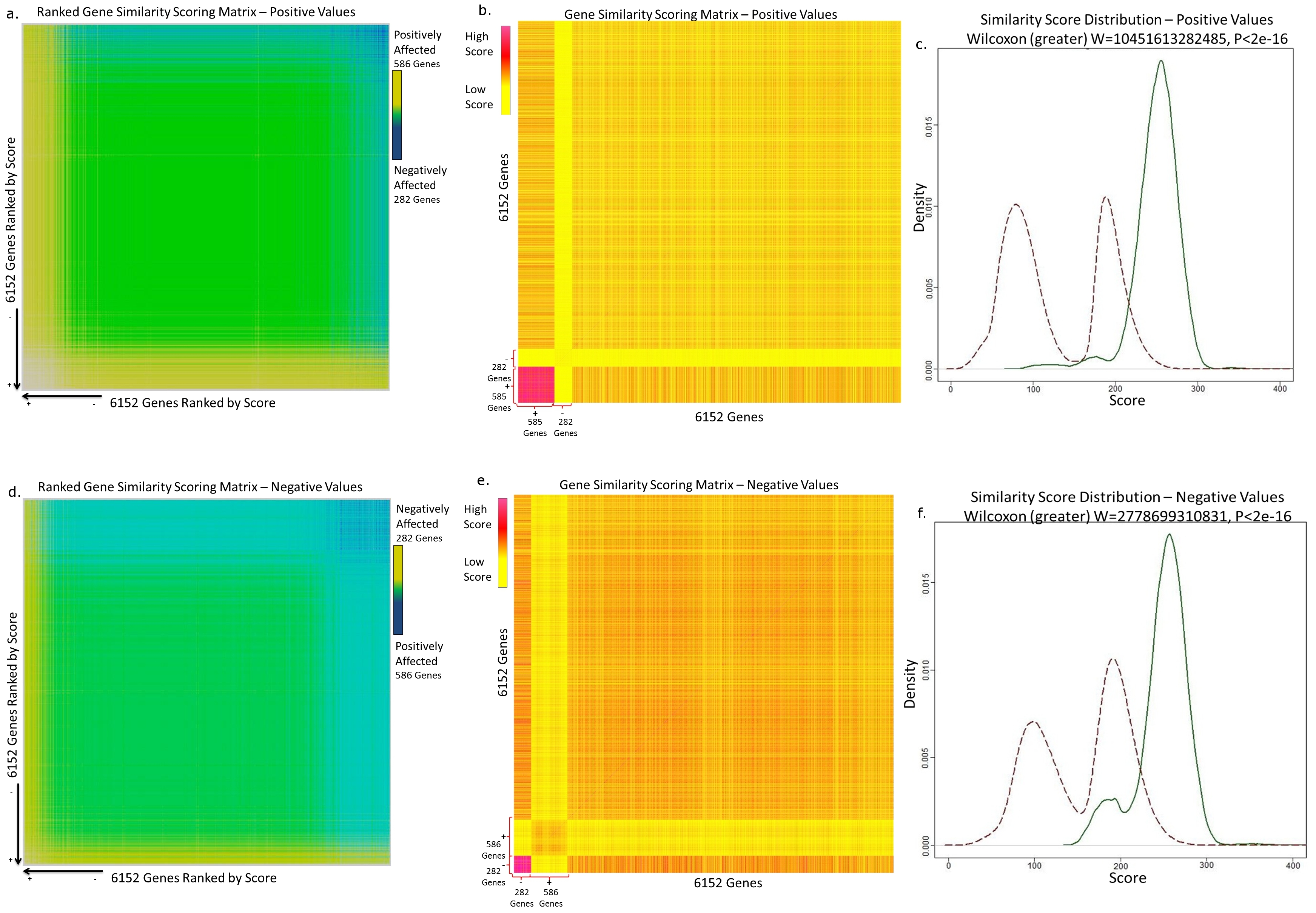

Supplement: Additional file 6: Figure S2 — Shabtai et al. Gene similarity results using BE on a Genomic Expression Dataset. In order to locate genes of interest, the BE method was executed on a dataset of yeast response to environmental changes. Because both negative values and positive values are meaningful, we created two datasets where one included all positive values (negative values were set to 0) and the second dataset included all negative values, set to their absolute value (positive values were set to 0). Results show how the BE method successfully located the most affected genes , according to measured transcript abundance, confirming the 586 positively affected genes (a), and the 282 negatively affected genes (d), marked in yellow in the ranked scores as seen as the exceedingly affected genes. The higher scores, that the 868 genes received compared to other genes, can be seen in light green for both positive (b) and negative (e) scores. The 868 genes received statistically significant greater scores than other genes both for positive (c P<2e-16) and negative (f P<2e-16) affected genes where the full green line represents the positively (c), induced genes (c), and negatively, repressed genes (f), and the fragmented red line represents the rest of the genes. The distribution of scores for the less affected genes displays two peaks due to lower scores for the negative genes compared to the other genes and seen as two dark stripes (b), marked in blue at the low end scores (a). [file 1471-2105-13-245-S6.jpg]

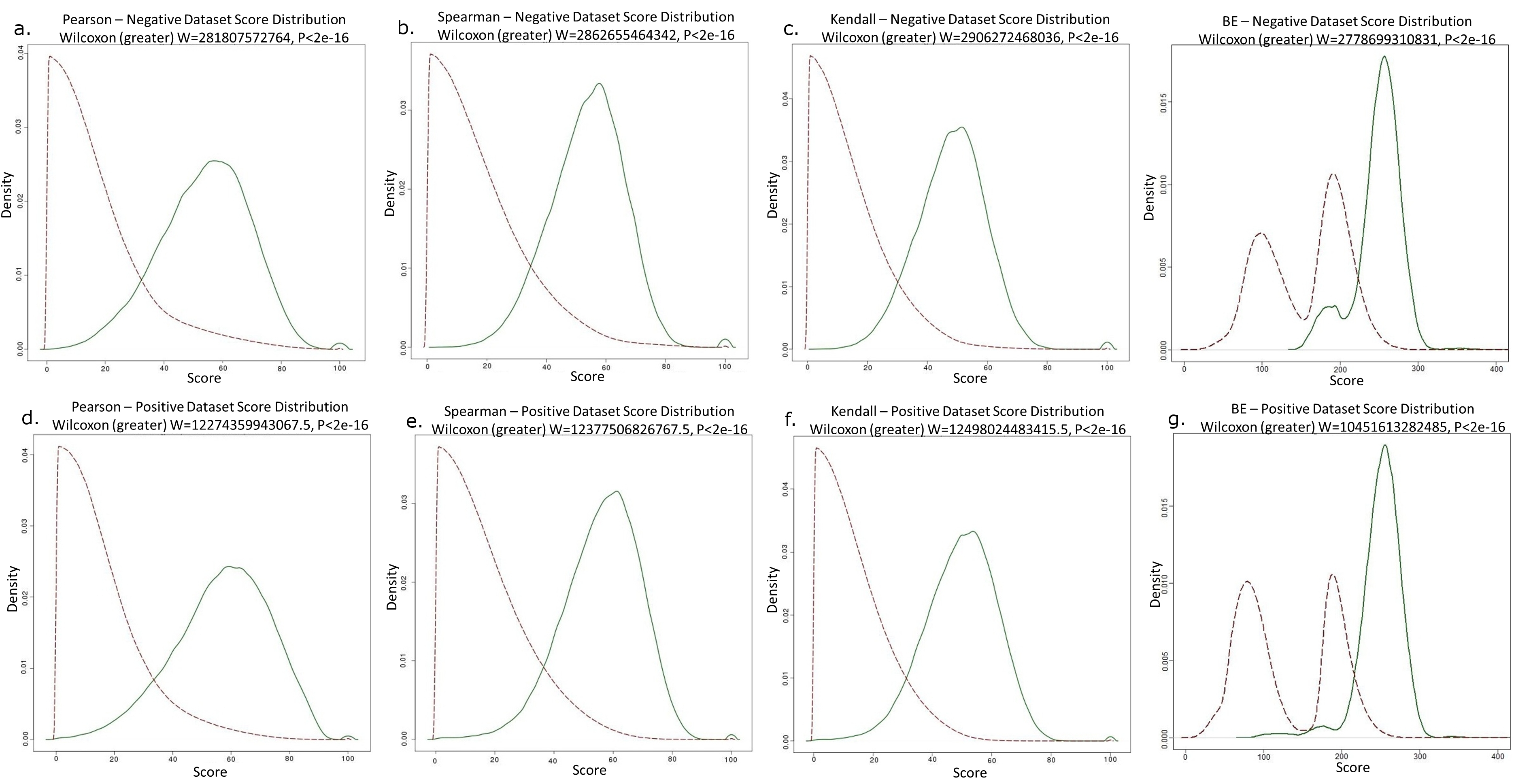

Supplement: Additional file 7: Figure S3 — Shabtai et al. Score distributions of several comparison methods for a Genomic Expression dataset. The distribution of scores of the Gasch et al. study dataset. The green line represents the score distribution of the previously reported group of genes found to be significantly affected by the stress treatments. For the negative score dataset (a, b, c, d), the green line represents the group of ~300 repressed genes, and for the positive score dataset (e, f, g, h), the green line represents the group of ~600 induced genes. The fragmented red line represents the score distribution of the genes other than the reported group of genes. The methods used for comparing the score distribution included BE, Pearson, Spearman and Kendall correlations. All methods showed there are statistically significant higher scores for the reported genes (similar W statistic value) successfully locating the affected genes. The BE method performed as well as other methods identifying the affected group of genes, moreover, it differentiated the lower results and identified anti-correlation between the two groups of ~300 and ~600 affected genes by showing two peaks for the lower scores. [file 1471-2105-13-245-S7.jpg]

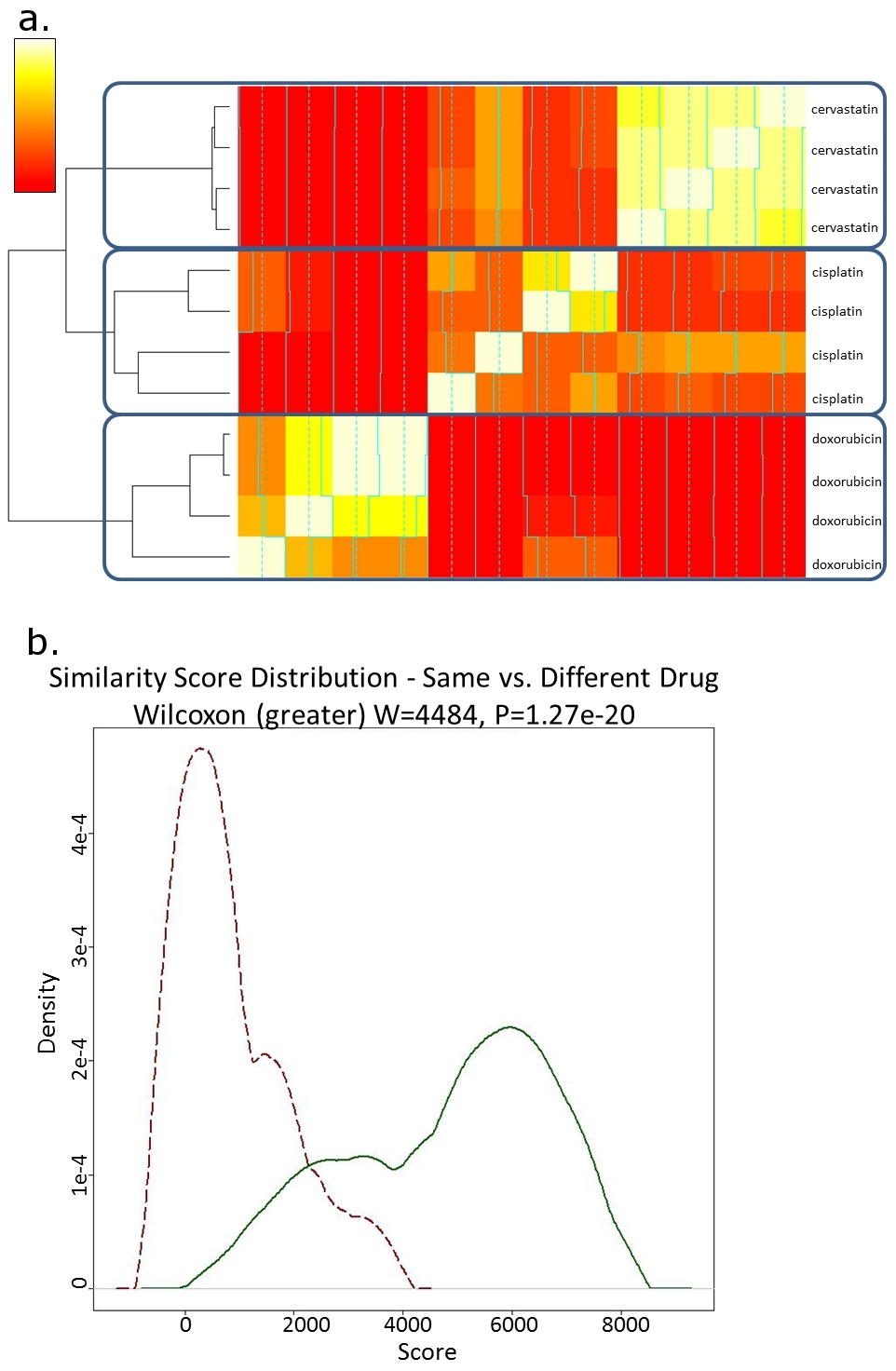

Supplement: Additional file 8: Figure S4 — Shabtai et al. Similarity results between experiments using BE on a sequencing dataset. Running the BE method on high throughput sequencing data successfully cluster experiments using the same drug (a). We used the Wilcoxon test to evaluate the distribution of the scores (b) of same drug experiment scores (green line) and different drug experiment scores (red line). These results showed that same drug scores received a statistically significant higher score than different drug scores (P=1.27e-20). [file 1471-2105-13-245-S8.jpg]

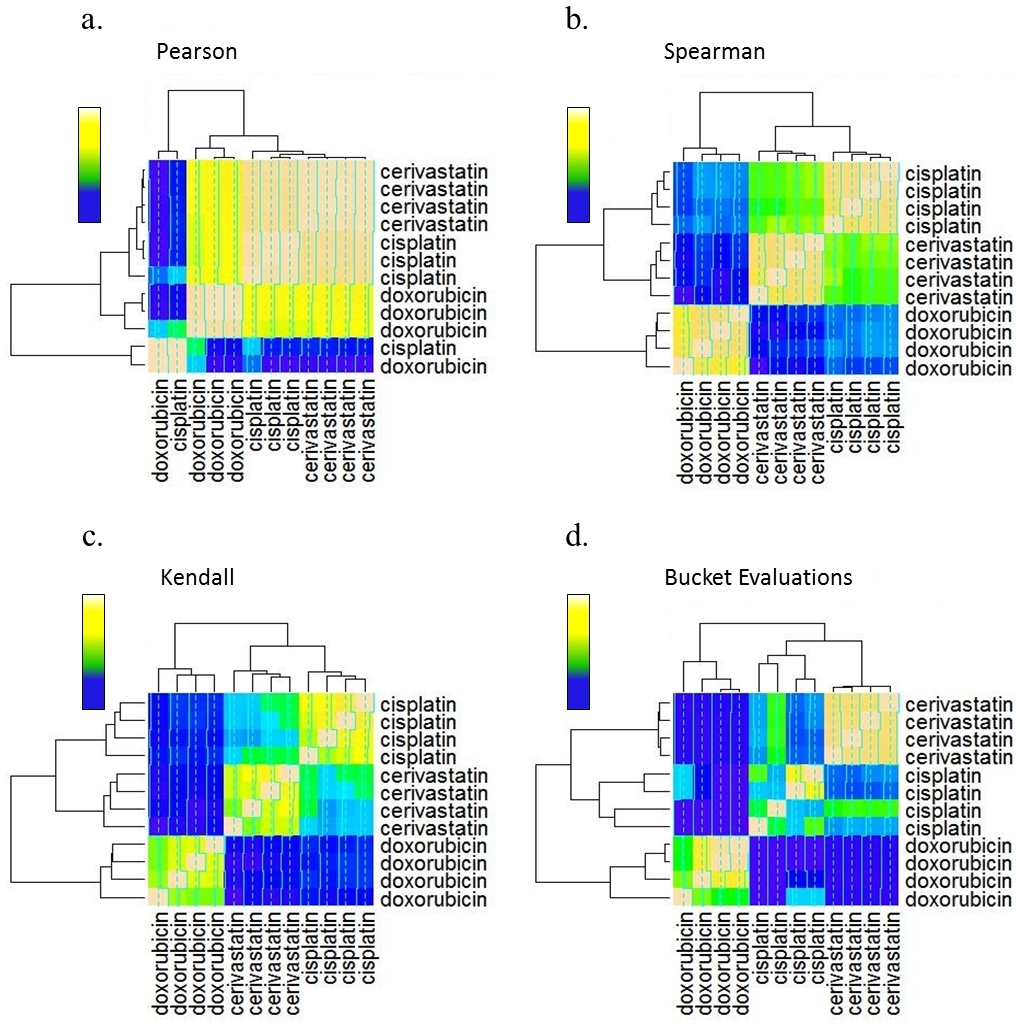

Supplement: Additional file 9: Figure S5 — Shabtai et al. Comparison of several correlation method outcomes using TAG4 Microarray dataset. A comparison of several methods, including Pearson (a), Spearman (b), Kendall (c) and BE (d), for finding correlations between barcode sequencing experiments. A heat-map and dendrogram displays the clustering of experiments for each method. For BE, Spearman and Kendall methods, all experiments that were performed using the same drug clustered together, showing BE (d) performed as well as other non-parametric methods, including Spearman (b) and Kendall (c). BE performed better than the Pearson correlation (a), where not all same-drug experiments clustered together. [file 1471-2105-13-245-S9.jpg]

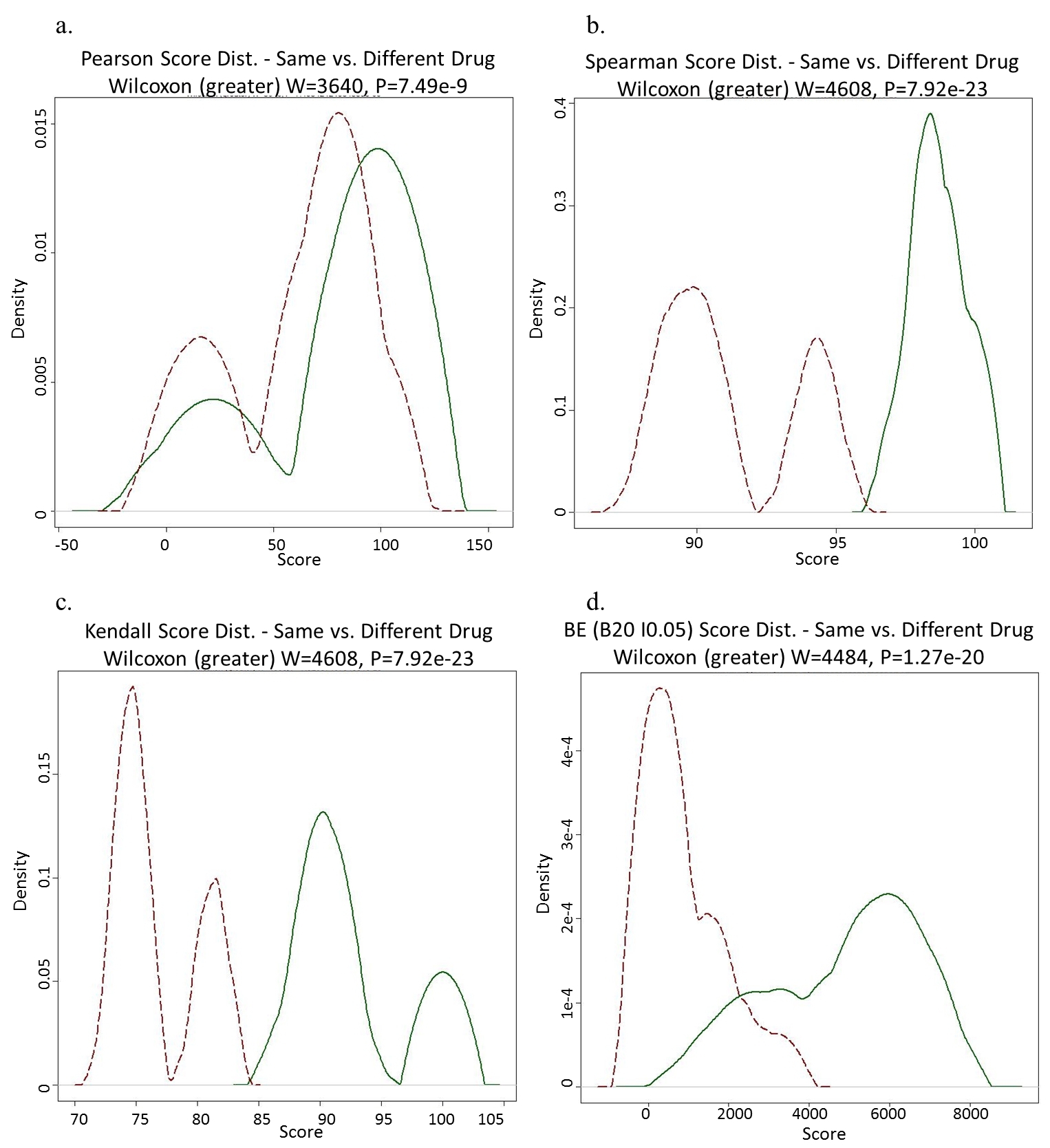

Supplement: Additional file 10: Figure S6 — Shabtai et al. Score distributions of several comparison methods for a sequencing dataset. The score distribution of several methods, including Pearson (a), Spearman (b), Kendall (c) and BE (d) of correlations scores of barcode sequencing experiments. The full green line represents the similarity score distribution of experiments performed using the same drug, while the fragmented red line represents the score distribution of experiments performed using different drugs. All methods present statistically significant greater scores to experiments performed using the same drug. [file 1471-2105-13-245-S10.jpg]

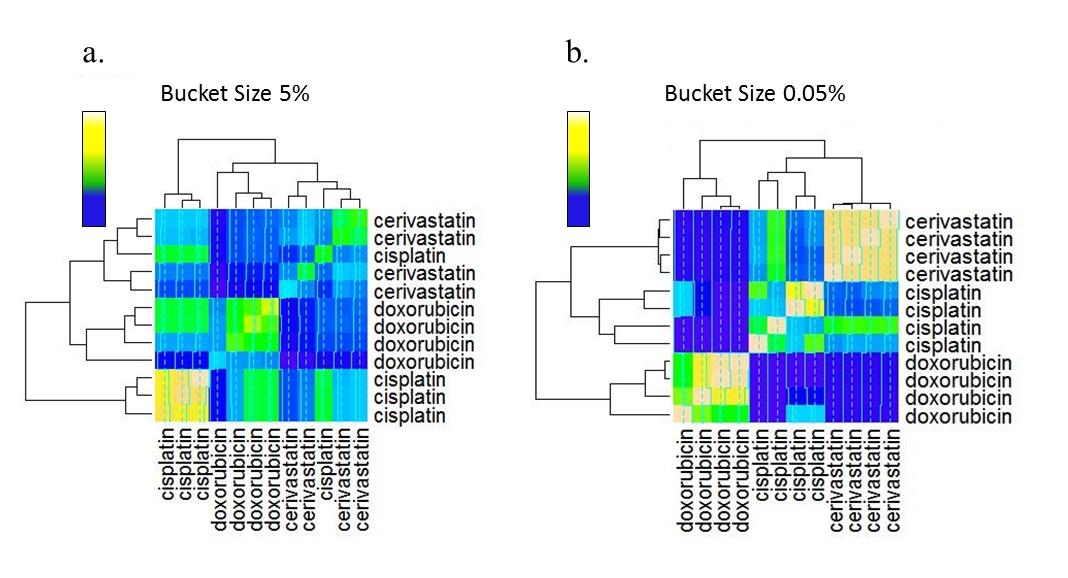

Supplement: Additional file 11: Figure S7 — Shabtai et al. Fine tuning the BE variable values. The output of using different BE variable values for high throughput sequencing dataset shows how fine tuning the value can produce a better result. When using an initial bucket size of 5%, not all experiments cluster according to the chemical compound (a). When using an initial bucket size of 0.05%, all experiments cluster according to the chemical compound (b), showing how fine tuning the value can produce better results. [file 1471-2105-13-245-S11.jpg]
