# Supplementary material for: An algorithm for chemical genomic profiling that minimizes batch effects: bucket evaluations
Source: BMC Bioinformatics. 2012 Sep 25;13:245. doi: 10.1186/1471-2105-13-245 (PMC3780717; doi:10.1186/1471-2105-13-245)
Supplement: Additional file 13 — Software Manual. An explanation of the software architecture, and how to use the software. [file 1471-2105-13-245-S13.pdf]

Bucket Evaluations
Software Supplementary

Daniel Shabtai - Nislow/Giaever Lab

© Copyright by Daniel Shabtai 2012

Bucket Evaluations
Software Supplementary

Daniel Shabtai - Nislow/Giaever Lab

Funded by:

Canadian Cancer Society / Société canadienne du cancer

CIHR IRSC
Canadian Institutes of Health Research / Instituts de recherche en santé du Canada

Canada Foundation for Innovation
Fondation canadienne pour l'innovation

Canada Research Chairs
www.chairs-chaires.gc.ca

genome.gov
National Human Genome Research Institute
National Institutes of Health

2012

Table of Contents

Table of Contents 3
Bucket Evaluations Software Supplementary 4
1.1. User Experience 7
1.2. Main GUI Window (MGUIW) 9
1.2.1. User Input 9
1.2.2. Status Notifications 13
1.2.3. Cancel Run 15
1.3. Information Form 15
1.4. BE Thread Manager (BETM) 16

Bucket Evaluations Software Supplementary

In order to create a program for running the BE algorithm, I took into consideration several design approaches, such as: (1) creating a program that allows the user to decide on resource allocation for better performance according to the hardware abilities, and (2) an easy-to-use graphical user interface (GUI) (Figure 1).

The program includes a multithreaded architecture that allows the GUI to remain active, while multiple threads are executing the analysis on the dataset provided (Figure 2). The design of the program consisted of 3 independent threads, including the *main GUI window* (MGUIW) thread, *information form* thread, and the *BE Thread Manager* (BETM). In addition to these threads are the dataset analysis threads. The amount of *Dataset Threads* (DT) is a variable set by the user (Figure 2).

Figure 1

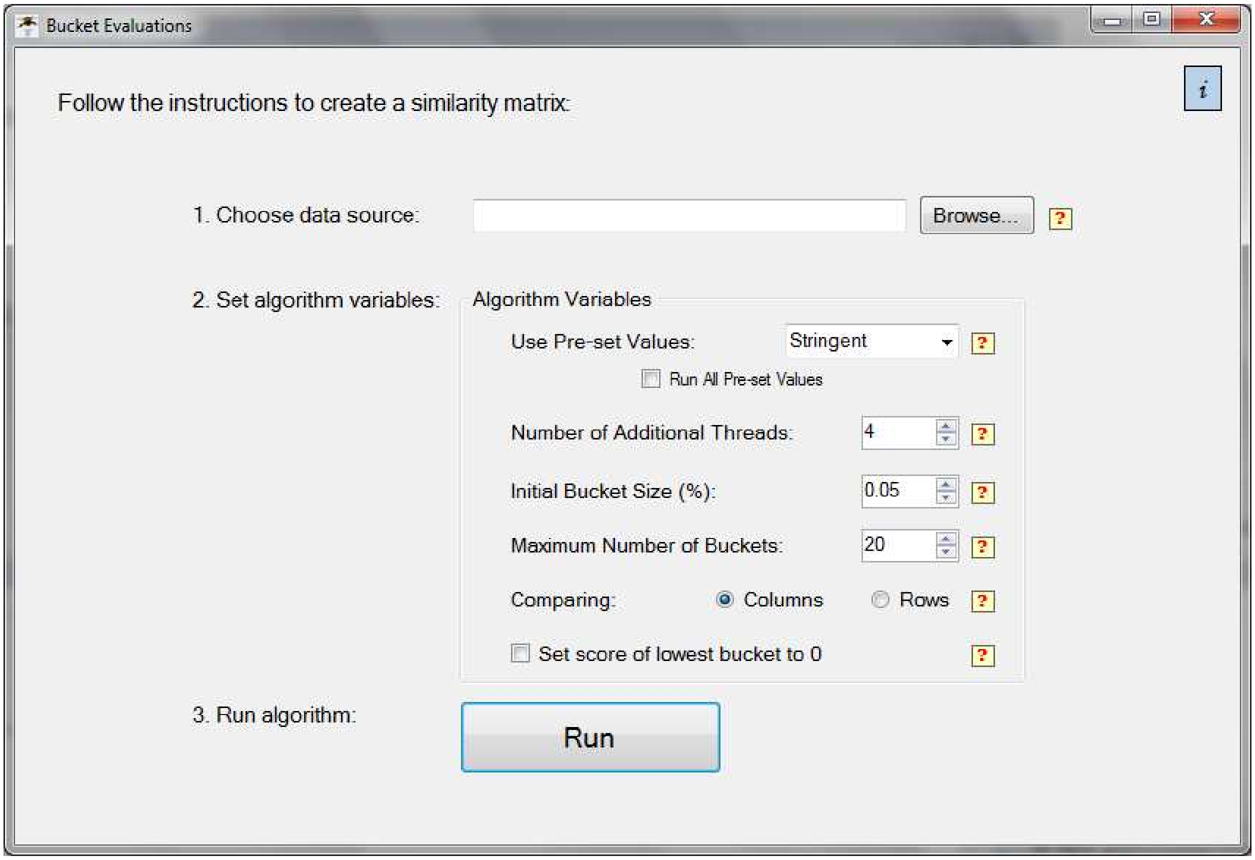

Figure 1 | Bucket Evaluations Software Graphical User Interface. This image is the main window, which displayed to the user. It provides the user the needed steps to load a dataset (step 1), run the BE algorithm and produce a similarity matrix as a file (step 3). This window gives the user an option to set the algorithm's variables (step 2), and provides help for each of the variables using a tool-tip hover button.

Figure 2

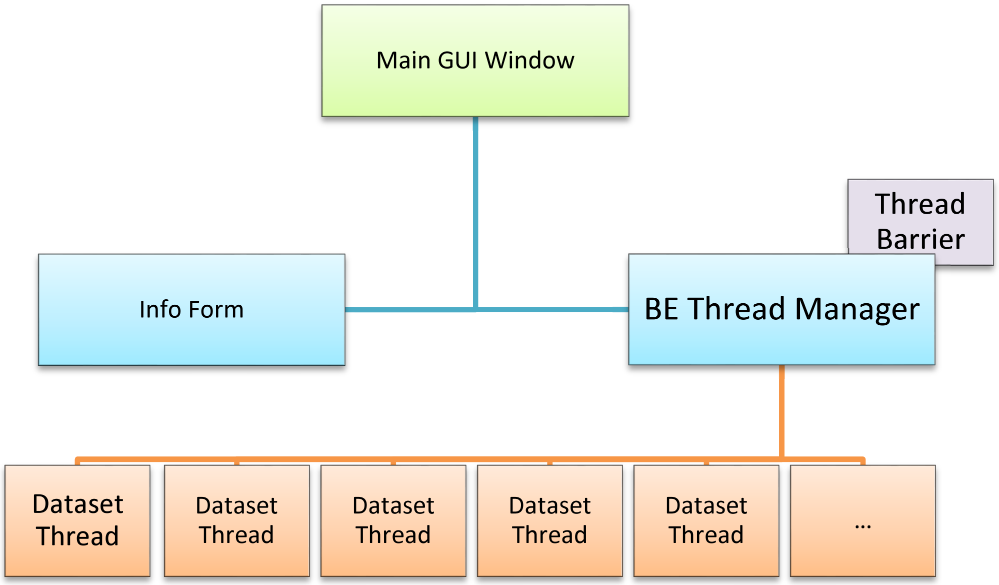

Figure 2 | Bucket Evaluations Software Architecture. Each rectangle represents a class in the program. The green blue and orange rectangles represent separate threads. The Thread Barrier (purple rectangle) is a separate object used by the BE Thread Manager.

1. User Experience

Running the analysis requires three steps, as described in the MGUIW (Figure 1):

1. Choose a data source file for which you wish to create a similarity matrix. Clicking the 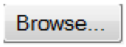

button opens a file browser for choosing the data source file (Figure 3). The data source file should be a standard tab delimited file, which includes the column names, row names and numeric data results.
2. Set algorithm variables according to the data type you are using. As previously mentioned, results may be more accurate by manipulating these values. It is therefore recommended to run the source dataset multiple times with different values. This will allow to fine tune the variables to best the user's data source.
3. Clicking the 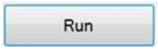

button opens a window requesting the user for a file save location (Figure 4). The program writes the similarity matrix information to the save location, which is the output target file. The program commences the analysis of the data once the user confirms the output location. If the dataset is in an incorrect format, or if there is any other problem with the run, a message will be displayed to the user using exception handlers, which are code sections that deal with faulty input.

Once the program commences the analysis, the status of the run is displayed to the user. The status is displayed by using a progress bar, percentage status, and status text components, which are constantly updated throughout the run.

Figure 3

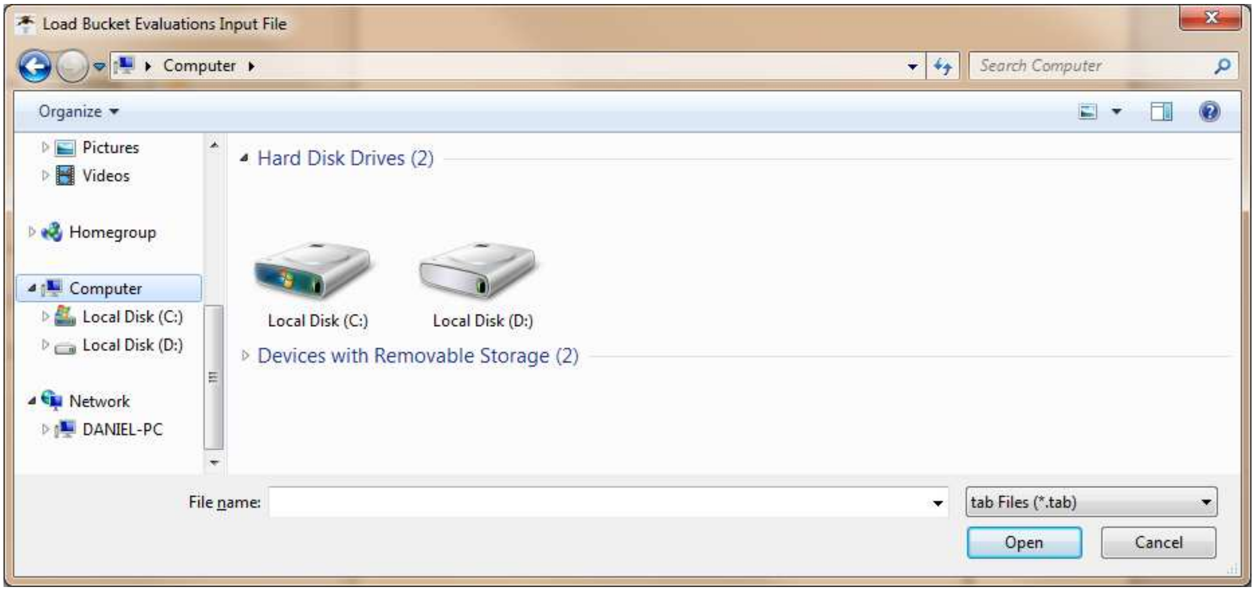

Figure 3 | Bucket Evaluations Software - Load file location window

Figure 4

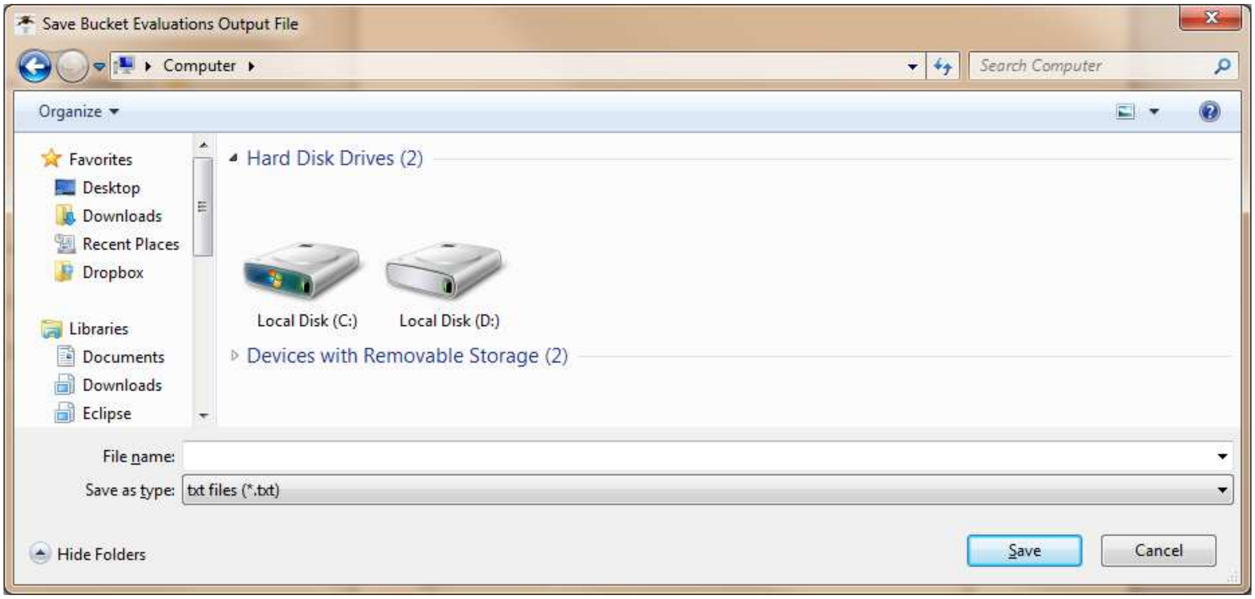

Figure 4 | Bucket Evaluations Software - Save file location window

2. Main GUI Window (MGUIW)

The MGUIW thread is the initial execution thread, and is responsible for user input and status notifications to the user. The user input includes input file selection window, initial input file validations, algorithm parameter input etc. (Figure 1). The MGUIW thread also includes tool-tip hover labels 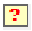

, which provide the user with a text explanation of the variable fields to fill.

User Input

Each input parameter consists of a GUI object that is relevant to the type of data needed (e.g. file location requires text and initial bucket size requires a number). The parameters, which the user can modify include:

- "Choose data source" – A variable input that consists of a textbox and a 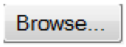

button components. Allows users to select the data file which they wish to load. The data must be a tab delimited file including column and row names. For example (file format: "\tC1\tC2\tC3\nR1\t1\t2\t3\nR2\t4\t5\t6\nR3\t7\t8\t9"):

	C1	C2	C3
R1	1	2	3
R2	4	5	6
R3	7	8	9

- "Use Pre-set Values" – A variable input that consists of a combo box component. Allows the user to choose pre-set values for the BE variables, including: "*Stringent*" - Score is

dependent on more accuracy between the ranks of values, and "*Broad*" - The group of top ranked values is larger. A large group of top ranked values produces a high score for more distant ranks in each bucket. These values are set while assuming there are ~6000 values to compare. The sizes vary for different sizes of datasets.

- "Number of Additional Threads" – A variable input that consists of a numeric up-down component. Increasing the number of threads allows the algorithm to run analysis concurrently. Concurrent running of the algorithm may result in a faster outcome. Thread performance is dependent on the computer's hardware; therefore increasing the number of threads may result in a delayed outcome (see section 1.6.1).
- "Initial Bucket Size (%)" – A variable input that consists of a numeric up-down component. Allows the user to select the size of the first bucket. This value is the percentage of the dataset size. For example, if there are 10000 values to compare, and the value of the initial bucket size is set to 0.05, then the first bucket, which holds the most significant values, will hold 5 top values (which are 0.05% of 10000). Following buckets will be larger in size (Table 2). A small value will result in fewer scores considered as top hits, resulting in a stringent result when comparing columns/rows. The value set for the initial bucket size affects the results of the algorithm, therefore it is recommended to run the algorithm several times, while using different sets of variables, for finding the ideal variable values (Figure 5). These values can also be changed by using the different options in the pre-set values combo-box.
- "Maximum Number of Buckets" – A variable input that consists of a numeric up-down component. Allows the user to select the algorithm's maximum amount of levels to divide each experiment. For example, when comparing fitness defects of genes, this

variable will represent the maximum number of groups the genes will be divided. A small value in this field will result in a reduced effect on the similarity score of the lower values in the dataset (Table 2).

- "Comparing Columns/Rows" – A variable input that consists of radio button components. Allows the users to select what analysis they wish to run on the dataset. For example, if the columns represent the experiments and the rows the represent the gene's fitness defect, then selecting the "Column" radio button will create an experiment similarity scoring matrix. For example, for an input file such as (file format: "\tC1\tC2\tC3\nR1\t1\t2\t3\nR2\t4\t5\t6\nR3\t7\t8\t9"):

	C1	C2	C3
R1	1	2	3
R2	4	5	6
R3	7	8	9

selecting 'Column' will result in a similarity scoring matrix of C1-C3 versus C1-C3, while selecting 'Rows' will result in a scoring matrix of R1-R3 versus R1-R3.

- "Set score of lowest bucket to 0" – A variable input that consists of a checkbox component. If checked, the score for the lowest buckets is set to 0. This results in giving a score of 0 similarity to the lowest bucket, which contains the lowest ranks, hence avoiding the low end of ranks. If left unchecked, the lowest ranks can cause a higher score of similarity, as it is included in the final similarity score.

Figure 5

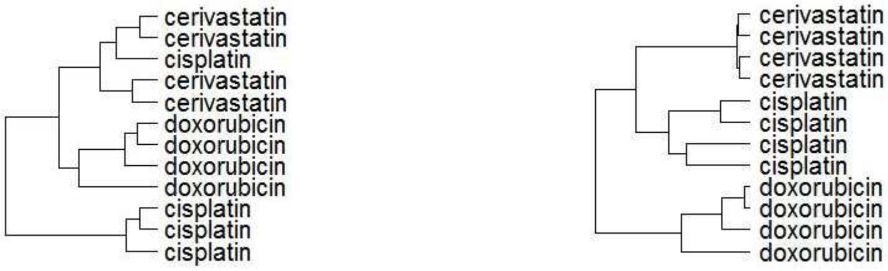

Figure 5 | Example of different result outputs for setting different bucket sizes when running the bucket evaluations algorithm. The dataset originated from high throughput sequencing (see section 3.4) with initial bucket sizes set to a broad value of 5% (a), and a stringent value of 0.05% (b). These dendrograms show the importance of running several parameter definitions for finding the best fit for the dataset. Both results show same drug treatments clustered together, though this dataset required a stringent set of variables as seen in dendrogram b, as all same chemical compounds clustered together, while for broad values of the variables, not all chemical compounds clustered together (cisplatin).

Status Notifications

In addition to the user input, the MGUIW is responsible for status notifications to the user. Status notifications are important as the user can get information regarding what stage of the program is running at each moment. In order to allow the BETM and DTs to change the components of the MGUIW, delegates execute MGUIW methods from external threads. These delegates provide an up-to-date status to the user by manipulating several components, such as:

- Progress Bar – Provides a graphical display of progress percentage (Figure 6B).
- Percentage Label – Provides a numerical display of progress percentage (Figure 6C).
- Status Text Label – Provides a brief text explanation of the current analysis action that is performed on the dataset (Figure 6D). Once the analysis is completed, the Status Text Label displays the output file location.

Figure 6

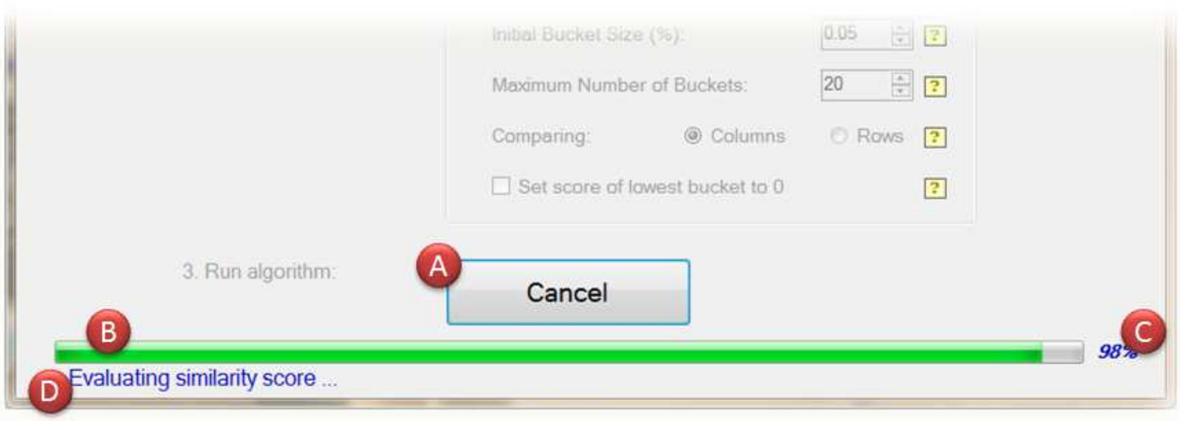

Figure 6 | Program GUI once executed. The user can cancel the run by clicking the “Cancel” button (A). This button is located at the same place the “Run” button was located prior execution. The status is presented to the user using a status bar (B), the current action percentage of the run (C), and text that provides a brief explanation of the current analysis action being performed (D).

Cancel Run

Once a run has been executed by clicking the 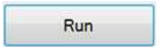

button, the MGUIW allows the user to cancel the run by clicking the 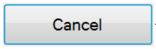

button (Figure 6A). The “Cancel” button is located at the same location that the “Run” button was located. Once “Cancel” is clicked, a series of events is initiated for terminating all running threads. The button-click event raises a flag that is periodically checked by the BETM. The raised flag leads to the following actions: (1) it prevents the creation of additional DTs by the BETM, (2) prevents existing DTs, that are in queue prior running, from starting analysis on the dataset, and (3) leaves the DTs, that are already running, to terminate upon completion.

3. Information Form

The *Information Form* is a separate thread, accessible through the 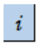

button on the MGUIW. It provides information about software version, and usage citation. This form is executed as a separate thread, therefore can also be displayed when the analysis is underway.

4. BE Thread Manager (BETM)

The BETM is a thread which controls the flow of the analysis according to the user's parameters, and orchestrates over multiple DTs. In order to do so, it uses thread control tools such as semaphores and thread barriers.

Semaphores are software objects that limit the amount of running threads. If the defined number of running threads is at its maximum, the semaphore puts any additional threads into sleep mode. Once a running thread is terminated, the semaphore activates one of the queued threads. The semaphores were used to limit the amount of DTs running at the same time. The amount of allowed threads is set by the user prior the run. If the user sets the number of additional threads to 0 (Figure 1), the analysis will be performed from the BETM thread and not from additional DTs.

The thread barrier is an object responsible for preventing a selected thread from running as long as a certain group of signal threads have not completed their run. The barrier prevents the selected thread from running, by putting it in sleep mode. Once the group of signal threads complete their run, the sleeping thread is awakened. The thread barrier was not part of .Net 3.0 framework, therefore, I implemented the barrier object as a separate class. The barrier was used for controlling the stages of the analysis, adding DTs to the queue only for relevant sections of the dataset. I also used the thread barrier for preventing the queue of DTs from becoming too large. Preventing an oversized DT queue is important for the event that the "Cancel" button is clicked, as there is a limited amount of threads in the queue that need to be cancelled.

Each part of the analysis is divided into sub-tasks that are performed by DTs. The BETM creates a limited amount of DTs so that if the user cancels the run, there is a limited amount of DTs

awaiting execution. Because the dataset is analysed by multiple threads, the BETM makes sure that the sections being analysed are not overrun by other threads, and therefore provides the DT with a mutually exclusive section for it to work on. These subtasks included tasks such as ranking scores of specific columns, entering the values of a specific column into buckets, and comparing experiments. For example, for a dataset of size 100X100 there will be ~10200 subtasks (100 ranks + 100 bucket definitions + ~10000 comparisons) assigned to threads. Once the analysis is completed, the BETM saves the output to a standard tab delimited file which is located in the path selected by the user.
